# eHealth Interventions to Address HIV and Other Sexually Transmitted Infections, Sexual Risk Behavior, Substance Use, and Mental Ill-health in Men Who Have Sex With Men: Systematic Review and Meta-analysis

**DOI:** 10.2196/27061

**Published:** 2022-04-06

**Authors:** GJ Melendez-Torres, Rebecca Meiksin, T Charles Witzel, Peter Weatherburn, Jane Falconer, Chris Bonell

**Affiliations:** 1 University of Exeter Medical School Exeter United Kingdom; 2 London School of Hygiene and Tropical Medicine London United Kingdom

**Keywords:** men who have sex with men, HIV and sexually transmitted infections, mental health, substance use, mobile apps, HIV, eHealth, electronic media, mobile phone apps, sexual risk

## Abstract

**Background:**

Men who have sex with men experience disproportionately high levels of HIV and other sexually transmitted infections (STIs), sexual risk behavior, substance use, and mental ill-health. These experiences are interrelated, and these interrelations are potentiated by structural conditions of discrimination, stigma, and unequal access to appropriate health services, and they magnify each other and have intersecting causal pathways, worsening both risk for each condition and risk for the negative sequelae of each condition. eHealth interventions could address these issues simultaneously and thus have wide-ranging and greater effects than would be for any 1 outcome alone.

**Objective:**

We systematically reviewed the evidence for the effectiveness of eHealth interventions in addressing these outcomes separately or together.

**Methods:**

We searched 19 databases for randomized trials of interactive or noninteractive eHealth interventions delivered via mobile phone apps, internet, or other electronic media to populations consisting entirely or principally of men who have sex with men to prevent HIV, STIs, sexual risk behavior, alcohol and drug use, or common mental illnesses. We extracted data and appraised each study, estimated meta-analyses where possible by using random effects and robust variance estimation, and assessed the certainty of our findings (closeness of the estimated effect to the true effect) by using GRADE (Grading of Recommendations, Assessment, Development and Evaluations).

**Results:**

We included 14 trials, of which 13 included active versus control comparisons; none reported mental health outcomes, and all drew from 12 months or less of follow-up postintervention. Findings for STIs drew on low numbers of studies and did not suggest consistent short-term (<3 months postintervention; *d*=0.17, 95% CI –0.18 to 0.52; I^2^=0%; 2 studies) or midterm (3-12 months postintervention, no meta-analysis, 1 study) evidence of effectiveness. Eight studies considering sexual risk behavior outcomes suggested a short-term, nonsignificant reduction (*d*=–0.14, 95% CI –0.30 to 0.03) with very low certainty, but 6 studies reporting midterm follow-ups suggested a significant impact on reducing sexual risk behavior (*d*=–0.12, 95% CI –0.19 to –0.05) with low certainty. Meta-analyses could not be undertaken for alcohol and drug use (2 heterogeneous studies) or for HIV infections (1 study for each of short-term or midterm follow-up), and alcohol outcomes alone were not captured in the included studies. Certainty was graded as low to very low for most outcomes, including all meta-analyses.

**Conclusions:**

To create a comprehensive eHealth intervention that targets multiple outcomes, intervention evaluations should seek to generalize both mechanisms and components that are successfully used to achieve change in 1 outcome over multiple outcomes. However, additional evaluations of interventions seeking to address outcomes other than sexual risk behavior are needed before development and evaluation of a joined-up intervention.

## Introduction

Men who have sex with men (MSM) experience HIV and other sexually transmitted infections (STIs), sexual risk behavior [1,2], substance use disorders [3-8], and mental ill-health at higher levels than the general population [9,10]. These experiences are interrelated, and these interrelations are potentiated by structural conditions of discrimination, stigma, and unequal access to appropriate health services, and they magnify each other and have intersecting causal pathways, worsening both risk for each condition and risk for the negative sequelae of each condition. This is known as a syndemic. MSM who report substance use are more likely to report condomless anal intercourse and HIV infection [11]. MSM reporting higher levels of anxiety and depression are more likely to have potential alcohol dependency [12], and MSM with depressive symptoms report more condomless anal intercourse [13]. Public health strategies to address these outcomes together therefore have the potential to achieve multiplicative effects.

eHealth interventions are those facilitated by electronic media and devices. Such interventions aim to promote healthy behaviors and mental health, for example, by challenging thought patterns that obstruct change, increasing or maintaining motivation, setting and reviewing goals, and providing feedback on behavior. eHealth interventions have the capacity to acceptably and cheaply address multiple risk behaviors and health states by assessing individual needs, identifying connections between these, and then tailoring support [14]. This has the potential for multiplicative effects, given the same individuals commonly experience multiple risk behaviors and health states where addressing on behavior or health state can predispose a change in others [15]. eHealth interventions targeting sexual health, substance use, and mental ill-health among MSM most commonly draw on the information-motivation-behavioral skills model and social cognitive theory, while also often drawing on other scientific theories of behavior and behavior change [16]. Systematic reviews focused on general or mixed populations report that eHealth interventions can reduce alcohol use [17] and address common causes of mental ill-health [18-24]. Emerging evidence also suggests that eHealth interventions might reduce drug use and sexual risk behavior. Given the clustered and interacting nature of these problems among MSM, evidence of the effectiveness of eHealth interventions for addressing each of these outcomes among MSM might suggest the value of an eHealth intervention that addresses these outcomes simultaneously and holistically. This intervention might also have multiplicative effects in ameliorating these syndemic conditions.

Our goal in undertaking this systematic review was to jointly consider the evidence for the effectiveness of eHealth interventions in addressing HIV and other STIs, sexual risk behavior, substance use, and mental ill-health separately or together in order to assess whether the evidence base supports the development of a single eHealth intervention for MSM for these syndemically related conditions.

## Methods

This was part of a larger evidence synthesis project also examining theories of change and implementation studies for eHealth interventions for MSM. Our systematic review was registered in PROSPERO (International Prospective Register of Systematic Reviews, CRD42018110317).

### Inclusion and Exclusion Criteria

We included randomized trials, without regard to comparator, of interactive or noninteractive eHealth interventions delivered via mobile phone apps, internet, or other electronic media (ie, electronic communication technology) and delivered to populations consisting entirely or principally of MSM. We also required interventions to address prevention of HIV, STIs, sexual risk behavior, alcohol and drug use, or common mental illnesses as outcomes representing, for example, incident cases of STIs and HIV, number of condomless sex partners, substance use frequency or prevalence, or symptomatology or diagnoses of anxiety or depression. We excluded (1) eHealth interventions merely facilitating one-off contact, (2) those addressing HIV self-testing, clinic attendance, or STI partner notification only, and (3) interventions delivered by human providers via electronic media. These interventions were excluded to better facilitate an understanding of the effectiveness of “fully eHealth” interventions delivered over a period of time on individual health behaviors rather than on the management of STIs—one-off health education interventions delivered via video or other opportunistic interventions.

### Study Search and Selection

To locate studies, we searched 19 databases in October and November 2018, updating searches in April 2020. We also searched 3 trial registers to identify ongoing recently published or otherwise unindexed trials alongside searches for grey literature. The full details of the databases and a sample search string are shown in Multimedia Appendix 1. We searched included studies’ reference lists and contacted subject experts. We deduplicated the retrieved references and uploaded these to EPPI-Reviewer (v 4.0, EPPI-Center). An inclusion criteria worksheet with guidance notes was prepared and piloted by 2 reviewers screening batches of the same 50 references. Where the 2 reviewers disagreed, they met to discuss this and if possible, reach a consensus, with recourse to a third reviewer if necessary. Once the inclusion criteria worksheet generated 95% agreement, each title and abstract was screened by 1 reviewer. Two reviewers assessed each full text that passed screening.

### Data Extraction and Appraisal

Two reviewers independently extracted data from outcome evaluations by using existing tools, meeting to discuss in cases of disagreement. We extracted data on basic study details (target population, study location, timing and duration, research questions, or hypotheses); methods (design, sampling and sample size, data collection, and analysis); and intervention description (timing and duration, program development, theoretical framework/logic model, content and activities, providers, details of any intervention offered to the control group), as well as allocation; sequence generation and concealment; measures, follow-up, and blinding; retention; and data on outcomes. Trials were appraised using the Cochrane risk of bias tool [25]. Where there was a risk of missing data from published reports affecting our analysis, we contacted authors wherever possible to request additional information. The risk of bias domains were used to inform GRADE (Grading of Recommendations, Assessment, Development and Evaluations) tables, which reflects the certainty of evidence for each outcome and time point. GRADE is a tool for researchers to describe how close the effect estimated in a meta-analysis is to the “true” effect of the intervention.

### Meta-analysis Methods

We narratively synthesized outcome evaluations by type of comparison and then by outcome, thereby grouping effect estimates by a follow-up duration after the intervention (short-term involving less than 3 months and medium-term involving 3 months up to 1 year). We defined type of comparison as either active versus control, where interventions were compared against a business-as-usual or no-treatment control, and active versus active, where interventions were compared against other eligible eHealth interventions. Where necessary, we rebased follow-up times using the stated intervention length but report in our narrative synthesis follow-up times as described in original reports. After converting effect estimates to standardized mean differences, we used a robust variance estimation meta-analysis with random effects to synthesize outcomes by follow-up time and then across all follow-up times and quantified heterogeneity by using I^2^. Robust variance estimation permits the inclusion of multiple effect sizes per study by accounting for nonindependence in standard errors. If an indication of substantial heterogeneity was determined with fewer than 3 studies (eg, study-level I^2^ greater than 50%), we did not present a pooled estimate by follow-up time or across follow-up times. We were unable to assess publication bias owing to the number of effect sizes in any 1 meta-analysis. We intended to undertake a network meta-analysis but were unable to do so owing to the low number and variable quality of studies.

## Results

**Figure 1:**
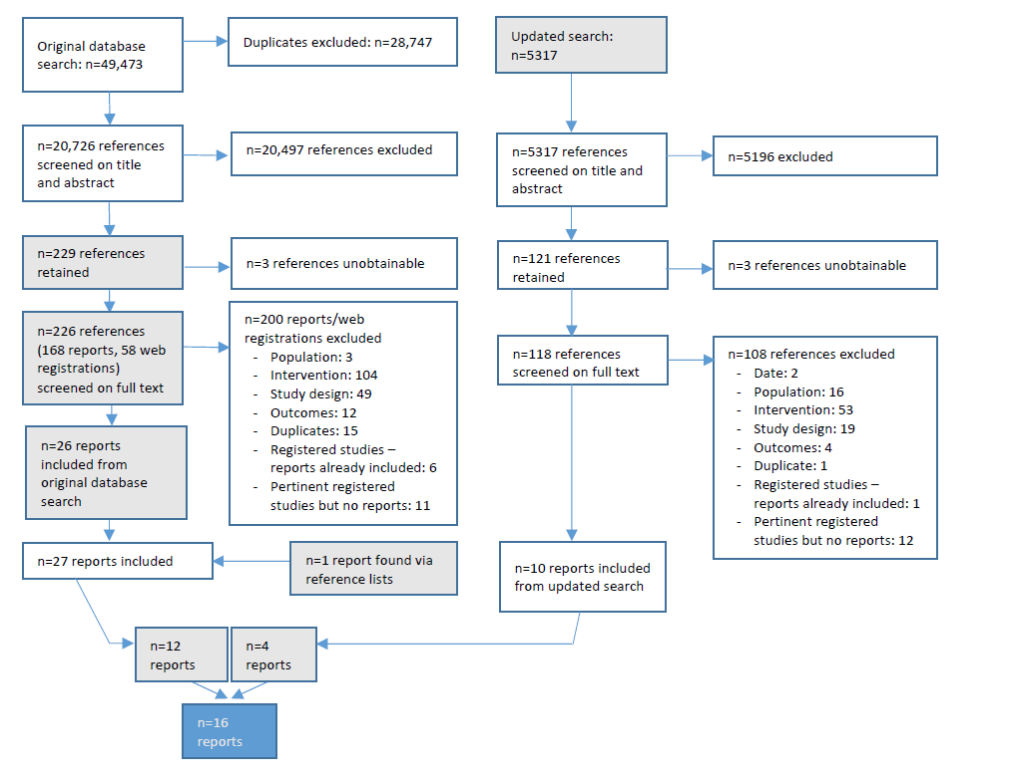
Study flowchart: outcome evaluations of eHealth interventions to address HIV and other sexually transmitted infections, sexual risk behavior, substance use, and mental ill-health in men who have sex with men.

Our original search yielded 49,473 references, with 20,726 remaining after deduplication (see Figure 1). We included a further 5317 references as a result of our updated search. From these, 344 references were not excluded on title and abstract and were screened on full texts over both waves of searching, yielding 16 records of 14 trials included in our syntheses.

### Included Trials and Interventions

The included trials [26-39] were published between 2006 and 2020 (see Multimedia Appendix 2). Of the included trials and included arms within those trials, 13 compared active intervention versus no treatment [26-38], while 2 [31,39] compared active interventions against each other. All but 4 trials were conducted in the United States: 1 was undertaken in the Netherlands [31], 1 in China [28], 1 in Taiwan [29], and 1 in Sweden [38]. We categorized interventions into time-limited or modular (guiding participants sequentially through intervention content from beginning to end; 11 trials) [26-28,30-32,34,35,37-39] and open-ended interventions (either user-responsive [36], static website content [33], or a multifeature app [29]; 3 trials). Interventions were generally delivered via website (including mobile website), with the exception of TXT-Auto [36], Safe Behavior and Screening (SBS) [29], and 1 downloaded videogame [30]. Within the 11 trials of time-limited or modular interventions, interventions had different foci. For example, myDEx [26] focused on comprehensive sexual education for young people, and China-Gates HIV Prevention Program [28], Hot and Safe M4M.org [27], Keep it Up! [34,35], Sexpulse [37], and WRAPP [38,39] presented general HIV prevention and sexual health content—all in interactive web-based modular formats—whereas SOLVE [30] used a videogame format. Davidovich et al [31] tested a tailored and a nontailored version of a web-based modular noninteractive intervention termed as cognitive vaccine, while Hirshfield et al [32] presented a modular video series, SexPositive. The 3 trials using open-ended interventions were delivered using text messages (TXT-Auto) [36], a mobile multifeature app (SBS) [29], or a static website [33].

### Quality of the Included Trials

**Figure 2:**
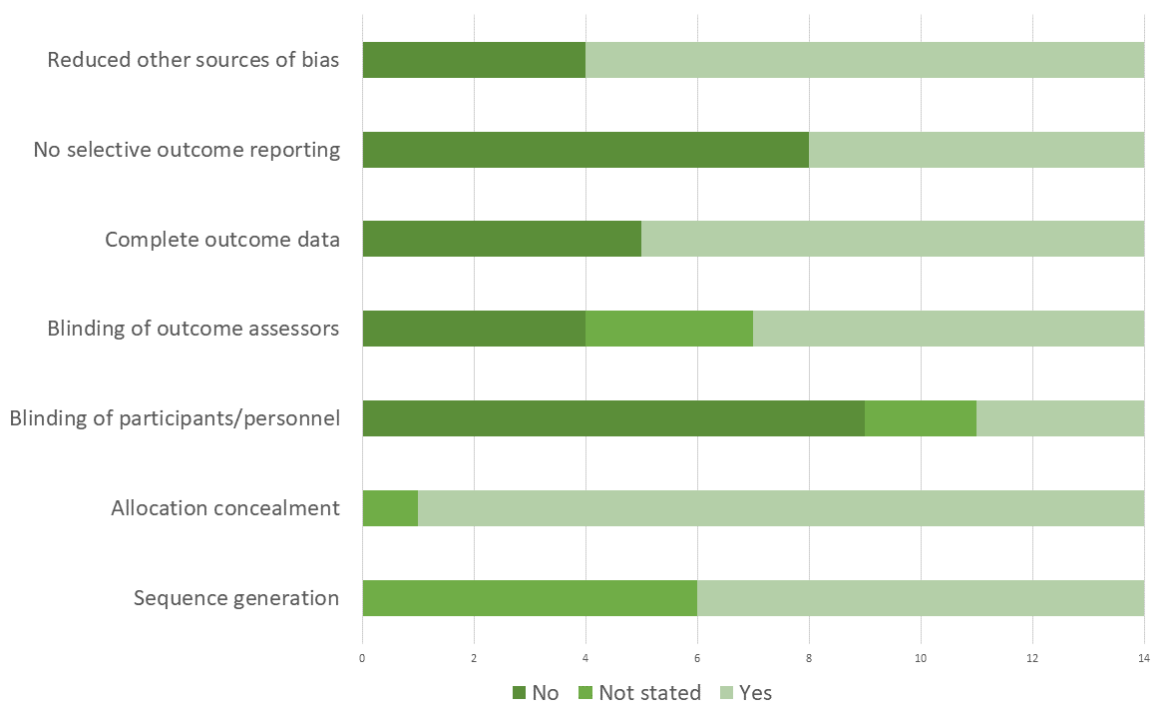
Summary risk of bias graph: outcome evaluations of eHealth interventions to address HIV and other sexually transmitted infections, sexual risk behavior, substance use, and mental ill-health in men who have sex with men.

We present in Figure 2 a summary risk of the bias graph, presenting review-level findings by domain. Trials generally had acceptable sequence generation and allocation concealment; however, blinding of outcome assessors was not always reported. Most trials presented complete outcome data, but 5 trials had high attrition, with imbalanced attrition between arms. Eight of the included trials appeared to be at high risk of bias owing to selective outcome reporting, including differences between reported outcomes and outcomes listed in the protocol. Study-level judgments and GRADE tables for each analysis are available in Multimedia Appendix 3.

### Mental Health

No included studies presented relevant mental health outcomes.

### Alcohol and Drug Use

Two studies [29,36] presented the estimates for drug use, both focused on open-ended interventions, but none included alcohol use specifically. Although both studies presented short-term results, only Reback et al [36] presented midterm results. Effect estimates in none of the follow-up categories suggested consistent evidence of effectiveness (see Table 1).

**Table 1:** Meta-analysis findings by outcome and follow-up time: eHealth interventions in men who have sex with men.

Outcome	Follow-up^a^	Results	Certainty
Alcohol and drug use	Short-term	No meta-analysis; 2 heterogeneous studies [29,36]	Very low
Alcohol and drug use	Midterm	No meta-analysis; 1 study [36]	Very low
HIV infections	Short-term	No meta-analysis; 1 study [29]	Low
HIV infections	Midterm	No meta-analysis; 1 study [35]	Very low
Sexually transmitted infections	Short-term	*d*=0.17, 95% CI –0.18 to 0.52, I^2^=0%, 2 studies [29,33]	Very low
Sexually transmitted infections	Midterm	No meta-analysis; 1 study included [35]	Moderate
Sexually transmitted infections	Overall	*d*=0.07, 95% CI –0.79 to 0.94, I^2^=16%, 3 studies [29,33,35]	N/A^b^
Sexual risk	Short-term	*d*=–0.14, 95% CI –0.30 to 0.03, I^2^=61%, 8 studies [26,27,29,32,34-37]	Very low
Sexual risk	Midterm	*d*=–0.12, 95% CI –0.19 to –0.05, I^2^=27%, 6 studies [28,31,32,35-37]	Low
Sexual risk	Overall	*d*=–0.15, 95% CI –0.26 to –0.05, I^2^=56%, 10 studies [26-29,31,32,34-37]	

^a^Short-term: up to 3 months postintervention; midterm: 3 months to a year after the intervention.

^b^N/A: Not applicable.

#### Short-term Results

After 6 months of app use (ie, at 6 months postrandomization), Chiou et al [29] found that the SBS open-ended intervention with general content reduced drug use as measured on a 5-point Likert scale (*β*=–1.19, SE 0.204, *P*<.001). In Reback et al [36], neither at the postintervention follow-up at 8 weeks postrandomization (*d*=0.15, 95% CI –0.16 to 0.45) nor at 3 months postrandomization (*d*=–0.03, 95% CI –0.34 to 0.28) was there evidence of a difference between TXT-Auto and the assessment-only condition on days of methamphetamine use. Statistical heterogeneity precluded meta-analysis of the results, and certainty of the assessment of evidence was graded as very low owing to risk of bias (details of randomization), inconsistency of studies, and imprecision of effect estimates.

#### Midterm Results

At 6 months postrandomization, there was no evidence of a difference between the TXT-Auto intervention tested in Reback et al [36] against the assessment-only condition on days of methamphetamine use (*d*=0.23, 95% CI –0.07 to 0.54). A similar estimate was produced at 9 months postrandomization (*d*=0.28, 95% CI –0.02 to 0.59). Although both estimates suggested a possible intervention effect of increased days of methamphetamine use in the intervention arm, authors noted that this could have been due to baseline imbalance.

### HIV Infections

Two studies presented findings relating to incident HIV infections, the former reporting on an open-ended intervention and the latter on a time-limited modular intervention [29,35]. In the short-term (at postintervention follow-up), Chiou et al [29] found an incidence rate ratio of 1.56 (95% CI 0.26-9.56) comparing SBS against a control group (see Table 1). In the midterm (at 6 months postintervention), Mustanski et al [35] found that incident HIV diagnoses were not different in the Keep It Up! intervention arm (9 diagnoses over 384 person-years) than in the control arm (8 diagnoses over 410 person-years). Included interventions did not have an overall impact on HIV infections, with an increase in incidence of HIV infections equivalent to 0.12 standard deviations but an imprecisely estimated confidence interval that included the point of no effect (95% CI –0.34 to 0.59). Certainty in the assessment of the evidence ranged from very low to low owing to risk of bias (selective outcome reporting) and imprecision of effect estimates.

### STIs

Three studies presented estimates for STIs: Chiou et al [29], Milam et al [33], and Mustanski et al [35]. Of these, Chiou et al [29] and Milam et al [33] presented short-term results on open-ended interventions, whereas Mustanski et al [35] presented midterm results on a time-limited/modular intervention. There was no evidence of short-term impacts on incident STIs, while there was some evidence, albeit from 1 study, for midterm impacts on incident STIs.

#### Short-term Results

At postintervention, Chiou et al [29] reported an incidence rate ratio of 1.39 (95% CI 0.31-6.37) for incident syphilis infections in the SBS group as compared to control. Milam et al [33] reported rates of any incident bacterial STIs (syphilis, gonorrhea, or chlamydia) over 12 months, which was the intervention period, finding that the proportions in each arm (30% intervention vs 25% control) were not statistically different (*P*=.50) and that the distribution of visits with new STIs per subject did not differ between arms (*P*=.57).

#### Midterm Results

Mustanski et al [35] reported results for several STIs, both individually and as a composite outcome, at 6 months postintervention. Findings were principally reported as risk ratio (RR) and suggested a statistically significant 40% difference in risk of any STI diagnosis (RR 0.60, 95% CI 0.38-0.95). Findings for individual STIs were not significant—specifically, urethral chlamydia (RR 0.60, 95% CI 0.13-2.34), urethral gonorrhea (RR 0.35, 95% CI 0.01-4.33), rectal chlamydia (RR 0.61, 95% CI 0.34-1.06), or rectal gonorrhea (RR 0.91, 95% CI 0.40-2.05). Another analysis of the outcome drew on a matched-pair analysis and estimated a within-subject reduction in risk of 68% for any STI (95% CI 0.40-0.83).

#### Meta-analysis

A meta-analysis of effect sizes with follow-up of less than 3 months (see Table 1) included 2 effect sizes from 2 studies reporting on open-ended interventions and suggested a nonsignificant increase in STIs in the intervention group as compared to that in the control group (*d*=0.17, 95% CI –0.18 to 0.52), with heterogeneity not meaningfully present in this meta-analysis (I^2^=0%) [29,33]. The certainty of evidence was very low due to risk of bias (details of randomization, selective outcome reporting) and imprecision of effect estimates. The overall analysis across short- and medium-term follow-up and intervention types drew on 3 studies contributing 7 effect sizes and suggested a small and nonsignificant increase in STIs in the intervention group as compared to the control group (*d*=0.07, 95% CI –0.79 to 0.94) and low heterogeneity (I^2^=16%) [29,33,35]. Plots are presented in Multimedia Appendix 4 [26-39].

### Sexual Risk Outcomes

Eleven studies presented estimates for sexual risk outcomes [26-32,34-36]. One further study [38] intended to present short-term results relating to sexual risk outcomes but did not estimate an effect owing to an unexpectedly low sample size. Sexual risk outcomes are organized into condomless anal intercourse (at the time of included studies’ publication usually referred to as unprotected anal intercourse), condom use, serononconcordant sex acts, and sex acts under the influence of drugs and alcohol.

#### Short-term Results

Nine studies presented short-term results [26,27,29,30,32,34-37]. Of these, Chiou et al [29] and Reback et al [36] report on open-ended interventions while the rest report on time-limited/modular interventions. Results are presented by type of outcome: condomless sex, condom use during sex, HIV serononconcordant sex, and sex under the influence of drugs.

**Figure 3:**
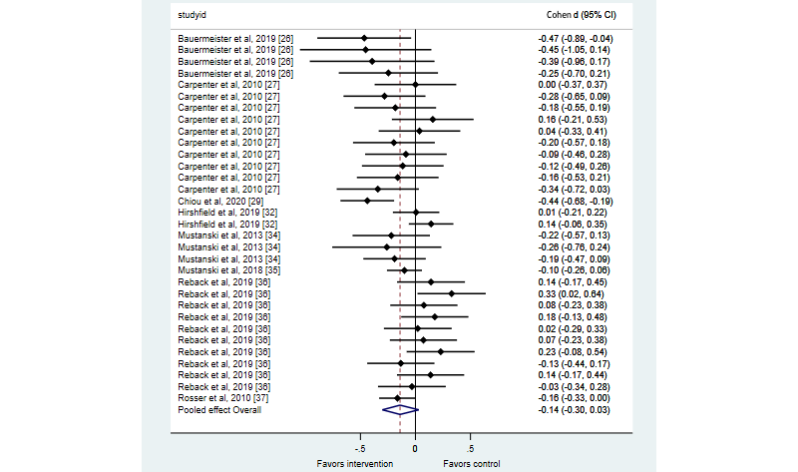
Short-term estimates of effects of eHealth interventions on sexual risk behaviors in men who have sex with men [26,27,29,32,34-37].

Six studies presenting short-term results for condomless sex yielded inconsistent evidence as to the effectiveness of interventions on this outcome [26,27,34-37]. All [26,27,34,35,37] but Reback et al [36] reported on time-limited/modular interventions. First, in their evaluation of the myDEx intervention, Bauermeister et al [26] found that at 90 days after the randomization (ie, at postintervention), intervention recipients had significantly lower odds than attention control recipients of any condomless receptive anal intercourse during the prior 3 months (odds ratio [OR] 0.43, 95% CI 0.20-0.94). The effect was lower in magnitude and nonsignificant for any condomless insertive anal intercourse (OR 0.64, 95% CI 0.28-1.44). However, at 3 months postbaseline, Carpenter et al [27] did not find that the Hot and Safe M4M intervention generated significant differences between groups on any condomless anal intercourse, condomless insertive anal intercourse, condomless receptive anal intercourse, condomless insertive oral intercourse, or condomless receptive oral intercourse. Specific significance tests per outcome were not provided, though calculated standardized mean differences (see Figure 3 for specific estimates) did not suggest a significant impact of the intervention. In the first evaluation of the Keep it Up! intervention, Mustanski et al [34] found that at 12 weeks postintervention, those who received the program had a lower rate ratio of condomless anal intercourse acts (rate ratio=0.56, *P*=.04; n=63). However, in the second evaluation of Keep it Up! [35], there was no significant difference between groups on any condomless anal intercourse, numbers of male condomless anal intercourse partners overall, or number of casual condomless anal intercourse partners at 3 months postrandomization, although specific significance tests were not reported for these outcomes. Calculated standardized mean differences between groups on reports of any condomless anal intercourse did not suggest a significant difference (*d*=–0.10, 95% CI –0.26 to 0.06). Rosser et al [37] estimated that the Sexpulse intervention reduced the number of male condomless anal intercourse partners by 16.8% at 3 months postbaseline, though this effect was only marginally significant (95% CI 0.691-1.000). Finally, reporting on an open-ended intervention, Reback et al [36] did not undertake endpoint-specific tests for condomless anal intercourse outcomes; however, we calculated that the intervention did not reduce episodes of condomless anal intercourse with main partners, anonymous partners, partners for transactional sex, or casual partners at 8 weeks or 3 months postbaseline (see Figure 3 for specific estimates). There was some suggestion of a harmful effect in terms of the intervention group having a higher number of condomless anal intercourse episodes with casual partners at 8 weeks postbaseline, but this may have been due to substantial baseline imbalance.

A study by Christensen et al [30] examined the mediating impact of shame on number of condomless anal intercourse events in the prior 3 months in the SOLVE time-limited/modular intervention at 3 months postbaseline. Estimates of the intervention’s total impact on condomless anal intercourse were not presented, but the significantly reported indirect effect on condomless anal intercourse through shame suggests a significant total effect of the intervention on condomless anal intercourse. However, these estimates are not directly comparable to the other tests of intervention impact presented here.

Two studies presented short-term results for condom use, and together, they reported mixed evidence of effectiveness on this outcome [29,34]. At 6 months postbaseline, Chiou et al [29] found that the SBS open-ended intervention increased the proportion of anal intercourse encounters where condoms were used by 20.7% (SE 0.058, *P*=.001) [29]. Similarly, in the first evaluation of the Keep it Up! time-limited/modular intervention, Mustanski et al [34] showed a reduced number of condom use errors (*d*=–0.19, *P*=.56) and condom failures (*d*=–0.22, *P*=.30), but not significantly.

Three studies presented short-term results for HIV serononconcordant sex acts and yielded mixed evidence on the effectiveness of time-limited/modular interventions for this outcome [26,27,32]. In their evaluation of the myDEx intervention, Bauermeister and colleagues [26] found that at 90 days postrandomization (ie, at postintervention), intervention recipients had lower odds than attention control recipients of any condomless receptive anal intercourse with serodiscordant or serounknown partners not known to be on pre-exposure prophylaxis or virally suppressed during the prior 3 months but not significantly so (OR 0.44, 95% CI 0.15-1.31); a similar pattern was found for insertive anal intercourse (OR 0.49, 95% CI 0.17-1.33). However, at 3 months postbaseline, Carpenter et al [27] found that the Hot and Safe M4M intervention generated greater reductions in all condomless anal intercourse events with partners of positive or unknown serostatus (group by time *F_1101_*=7.59, *P*=.007), including condomless insertive anal intercourse (group by time *F_1101_=*7.24, *P*=.008) but not condomless receptive anal intercourse (group by time *F_1101_=*1.35, *P*=.25). The intervention group also reported reduced condomless insertive oral intercourse events (group by time *F_1101_=*7.45, *P*=.007) and reduced condomless receptive oral intercourse events with partners of positive or unknown serostatus (group by time *F_1101_=*8.45, *P*=.004). Hirshfield et al [32] found that at 3 months postbaseline, the SexPositive intervention did not reduce either the number of condomless anal intercourse partners known to be serodiscordant (adjusted standardized *β*=.003, 95% CI –.168 to .178) or the number of condomless anal intercourse partners not known to be seroconcordant (adjusted standardized *β*=–.073, 95% CI –.332 to .051). Reback et al [36] was the only study to present short-term results for this category of sexual risk outcomes, reporting on an open-ended intervention. At neither 8 weeks postbaseline (*d*=0.23, 95% CI –0.08 to 0.54) nor 3 months postbaseline (*d*=0.08, 95% CI –0.23 to 0.38) was there a significant difference between groups on episodes of sex while on methamphetamine.

#### Midterm Results

Six studies presented midterm results—all but the last focused on time-limited/modular interventions [28,31,32,35-37]. Results are presented by type of outcome: condomless sex, condom use, serononconcordant sex acts, and sex acts under the influence of drugs.

**Figure 4:**
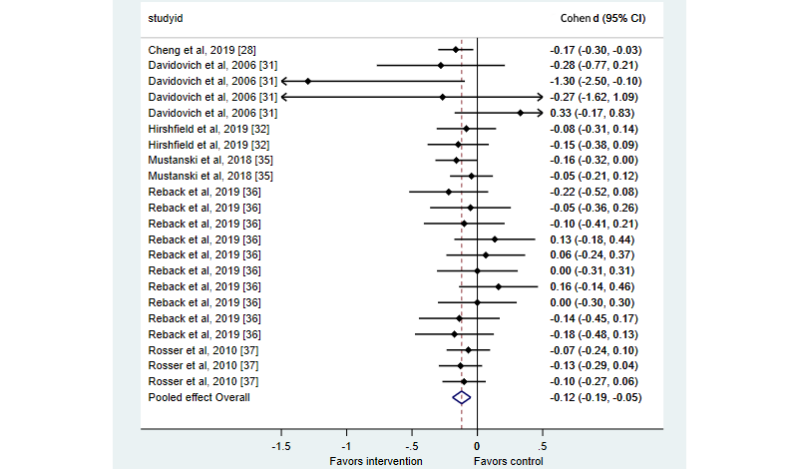
Midterm estimates of effects of eHealth interventions on sexual risk behaviors in men who have sex with men [28,31,32,35-37].

Four studies presenting midterm results for condomless sex yielded inconsistent evidence as to the effectiveness of interventions on this outcome [28,35-37]. Let us first consider time-limited/modular interventions. First, in the evaluation of the China-Gates HIV Prevention Program, intervention participants were less likely than control participants at 6 months postbaseline to report condomless anal intercourse in the last 3 months with a risk difference of 9.3% (95% CI 1.1-17.5); estimates using multiple imputation to include the entire sample generated a similar estimate (8.9%, 95% CI 1.2-16.6) [28]. In the second evaluation of Keep it Up!, there was no significant difference between groups on numbers of male casual condomless anal intercourse acts and number of condomless anal intercourse partners at 6 or 12 months postrandomization, though specific significance tests were not reported for these outcomes [35]. However, at 12 months after the randomization, intervention participants were 17% less likely to report any condomless anal intercourse in the prior 3 months (95% CI 0.70-0.99). Rosser et al [37] estimated that the Sexpulse intervention did not reduce the number of male partners with condomless anal intercourse at 12 months postbaseline (incidence rate ratio 0.998, 95% CI 0.952-1.046). We also calculated that there was no evidence of a significant effect at 6 months (*d*=–0.13, 95% CI –0.29 to 0.04) or 9 months (*d*=–0.10, 95% CI –0.27 to 0.06) postbaseline. Regarding open-ended interventions, Reback et al [36] did not undertake endpoint-specific tests for condomless anal intercourse outcomes; however, we calculated that the intervention did not reduce episodes of condomless anal intercourse with main partners, anonymous partners, partners for transactional sex, or casual partners 6 months or 9 months postbaseline (see Figure 4 for specific estimates).

Studies of this outcome focused on time-limited/modular interventions only. Davidovich et al [31] presented midterm results for condom use with no evidence of effectiveness, whereas this study and that by Hirshfield et al [32] presented inconsistent evidence of intervention effectiveness on midterm results for serononconcordant sex acts. In Davidovich et al [31], intervention participants receiving the tailored intervention were significantly more likely than control participants to practice negotiated safety (seroconcordant condomless anal intercourse or no condom use only in the context of monogamous relationships) as compared to condomless anal intercourse with partners of unknown HIV concordance if they received the tailored intervention (OR 10.50, 95% CI 1.19-92.72); however, intervention participants receiving the nontailored intervention did not show a significant difference on this outcome as compared to control recipients (OR 1.62, 95% CI 0.14-19.07) [31]. In this same study, intervention participants were not significantly different in the odds of condom use as compared to the control participants at 6 months postbaseline. This was the case comparing either the tailored version of the intervention (OR 1.66, 95% CI 0.68-4.02) or the nontailored version of the intervention (OR 0.55, 95% CI 0.22-1.37) against control, with OR values greater than 1, suggesting increased condom use. However, it should be noted that in this analysis, negotiated safety, condom use, and other condomless anal intercourse were mutually exclusive categories estimated as part of a multinomial regression model. In addition, Hirshfield et al [32] found that at 12 months postbaseline, the SexPositive intervention did not significantly reduce either the number of condomless anal intercourse partners known to be serodiscordant (adjusted standardized *β*=–.073, 95% CI –.332 to .051) or the number not specifically known to be seroconcordant (adjusted standardized *β*=–.084, 95% CI –.399 to .045). Reback et al [36] was the only study to present medium-term results for this category of sexual risk outcomes, reporting on an open-ended intervention. We calculated the differences by using endpoint means. At neither 6 months postbaseline (*d*=–0.10, 95% CI –0.41 to 0.21) nor 9 months postbaseline (*d*=–0.18, 95% CI –0.48 to 0.13) was there a significant difference between groups on episodes of sex while using methamphetamine.

#### Meta-analysis

The effect sizes for sexual risk outcomes are presented in Table 1 as well as in Figure 3 for outcomes at follow-ups of less than 3 months postintervention (short-term) and in Figure 4 for outcomes between 3 months and 1 year after the intervention (midterm). In both plots, negative effect sizes represent benefits. A meta-analysis drawing on 32 effect sizes from 8 studies with less than 3 months follow-up [26,27,29,32,34-37] found a suggestion of effectiveness, albeit not statistically significant (*d*=–0.14, 95% CI –0.30 to 0.03) with substantial heterogeneity (I^2^=61%). The certainty of evidence for this meta-analysis was graded as very low owing to risk of bias (details of randomization, selective outcome reporting), inconsistency of studies, and publication bias arising from the noninclusion of 2 studies [30,38]. A meta-analysis drawing on 22 effect sizes from 6 studies with 3 months to 1 year of follow-up suggested a significant impact on reducing sexual risk (*d*=–0.12, 95% CI –0.19 to –0.05) but with low heterogeneity (I^2^=27%). The certainty of evidence for this meta-analysis was graded as low owing to risk of bias (details of randomization, selective outcome reporting). We then pooled estimates regardless of follow-up time. Based on 54 effect sizes from 10 studies [26-29,31,32,34-37], interventions significantly reduced the sexual risk as compared to control groups (*d*=–0.15, 95% CI –0.26 to 0.05). This meta-analysis had substantial heterogeneity (I^2^=56%). To explore this heterogeneity, we compared interactive interventions [26-29,34-37] with noninteractive interventions [31,32]. A random-effects meta-regression did not suggest that this characteristic accounted for heterogeneity, with noninteractive interventions not meaningfully worse than interactive interventions (*β*=.12, 95% CI –.66 to .89).

### Active Versus Active Comparisons

Two included studies included comparisons between active interventions, which were time-limited/modular in approach [31,39]. Both studies reported sexual risk outcomes only, and only Davidovich et al [31] presented extractable outcome data. Thus, meta-analysis was not undertaken. In Davidovich et al [31], we calculated that participants receiving the tailored intervention were more likely than participants receiving the nontailored intervention to report, at 6 months postbaseline, the practice of negotiated safety (defined above) as compared to condomless anal intercourse with other partners (OR 6.50, 95% CI 2.49-16.90) and the practice of condom use as compared to condomless anal intercourse (OR 2.98, 95% CI 1.74-5.12). In their evaluation of time-limited interactive web-based modular interventions, Bowen et al [39] tested the impact of ordering modules with content about HIV knowledge, content about risk in casual or new partnerships, and content about contexts of risk on the proportion of anal intercourse partners with whom a condom was used every time. At postintervention, there was no statistical difference between modules on sexual risk, but specific group differences were not presented.

## Discussion

### Summary of Findings

Our systematic review of intervention effectiveness included 14 trials, of which 13 included active versus control comparisons. Trials included substance use, HIV, and STIs, and sexual risk behavior outcomes but not mental health outcomes. Substance use outcomes did not include alcohol use. Furthermore, all outcome estimates drew from 12 months or less of follow-up postintervention. A further 2 trials presented active versus active comparisons [31,39]. Neither trial found a difference between tested interventions on sexual risk outcomes and thus are not discussed further. Moreover, equity-relevant characteristics, for example, moderation of intervention effectiveness by income, ethnicity, and other social variables were not meaningfully addressed by this body of evidence.

In active versus control comparisons, findings for drug use drew on 2 studies in short-term follow-up [29,36], which could not be meta-analyzed owing to extreme heterogeneity, and 1 study in midterm follow-up [36]. Together, these studies did not present consistent evidence of effectiveness, and the GRADE profile for both analyses suggested the certainty of evidence was very low. Analysis for HIV infection also drew on 2 studies—1 in short-term follow-up [29] and 1 in midterm follow-up [35]. Neither study suggested that an eHealth intervention was effective at reducing infections, though short follow-up times and low event rates precluded meaningful comparison. Again, the GRADE profile suggested that the certainty of these findings was low or very low. Analyses for STIs were similarly scant, drawing on 2 trials in the short-term [29,33] and 1 trial in the midterm follow-up [35]. Although a pooled analysis of short-term follow-ups suggested no impact of the interventions on incident STIs with very low certainty, the 1 trial informing the midterm follow-up did suggest a meaningful and statistically significant reduction in incident STIs, with corresponding moderate certainty [35].

The largest analyses assessed sexual risk behavior outcomes. Although the GRADE profile suggested that the certainty of the conclusions was very low or low owing primarily to the risk of bias in the included trials and possible publication bias, pooled estimates from midterm follow-ups drawing on 6 trials suggested a small and statistically significant impact of eHealth interventions in reducing the sexual risk behavior (*d*=–0.12). Pooled estimates from short-term follow-ups drawing on 8 trials did not reach statistical significance but suggested a trend toward reductions in sexual risk behavior of similar magnitude (*d*=–0.14). We tested whether the interactivity of the interventions was related to intervention effectiveness on sexual risk behaviors; however, a meta-regression did not suggest significant differences between interventions on the basis of this characteristic.

### Implications

The quality and quantity of evidence supporting the effectiveness on eHealth interventions was low or, in the case of mental health outcomes, nonexistent for most of the outcomes we set out to analyze. Even where meta-analyses drew on multiple studies, issues with included trials precluded certainty in the evidence presented. Moreover, despite substantial heterogeneity in meta-analyses for sexual risk behavior outcomes, we were unable to explain this heterogeneity. Another key gap in this systematic review related to the inclusion of outcomes that accurately reflect current knowledge about minimizing sexual risk. For example, a focus on condom use does not reflect that risk for HIV can be managed through effective biomedical means such as adherence to HIV treatment for people living with HIV or pre-exposure prophylaxis. It is likely that interventions designed in the current context would more explicitly acknowledge biomedical approaches to managing risk.

One of the questions we set out to address in this systematic review was if the existing evidence overall suggests that our scoped outcomes could coherently, feasibly, and effectively be addressed by a single health intervention. It is clear based on the meta-analyses presented that the evidence does not, as yet, suggest that this is the case, despite the consistent acceptability of these interventions we documented in a linked systematic review of process evaluations [40]. This is largely because interventions, with few exceptions, were focused on individual outcomes as well as the patchy effects for outcomes that were assessed. Only 2 interventions captured substance use outcomes alongside sexual risk behavior outcomes [29,36], where trials otherwise reported outcomes over multiple categories; this was between sexual risk behavior and either HIV or other STIs. No study reported outcomes for depression or anxiety, despite poor mental health being a key syndemic condition nor did any study report outcomes relating to alcohol use, despite this being the most commonly misused substance in high-income country settings.

In order to remedy this, future trials of eHealth interventions should include several considerations. First, given the complete lack of evidence in this area, trials should consider how to address poor mental health in MSM, with a focus on how determinants of poor mental health in MSM both relate to outcomes considered here (sexual ill-health, substance use) and other antecedents (eg, stigma). Second, trials should address a range of substance use behaviors that are syndemically linked to other relevant outcomes (mental well-being and sexual health), including alcohol use. Third, trials should incorporate follow-ups long enough and sample sizes large enough to detect a meaningful impact on HIV and other STIs, given the time needed to detect meaningful differences in HIV infection incidence. Alternatively, given the challenge associated with attaining sample sizes large enough to demonstrate impacts on STIs, studies could include a wider range of behavioral indicators closely linked with reduced STIs to improve confidence that a distal improvement in incident infections is likely. Most included studies reported only 1 or 2 relevant outcomes relating to sexual risk outcomes. Fourth, trials should ensure that interventions are not inequality-generating and that this is examined empirically. This is important because interventions may unintentionally exacerbate inequalities within groups due to, for example, differential access to mental or sexual health services or substance use services. Finally, to generate a joined-up comprehensive eHealth intervention that targets multiple outcomes, intervention evaluations should seek to generalize both mechanisms and components that are successfully used to achieve change in 1 outcome over multiple outcomes and should draw on current technologies and modalities. For example, a videogame download for a desktop computer is unlikely to be as acceptable as a specific app-based on text message–based intervention. The majority of the interventions used websites, but it was frequently unclear based on intervention descriptions how relevant these interventions would be in the current mobile health environment. It remains important to note that this is a rapidly developing field of research, and it is likely that a range of “next-generation” eHealth interventions will be evaluated in the near future. This will be valuable to understand how more recent technologies, including greater use of apps as opposed to websites, can support reducing health inequalities in MSM.

### Limitations

Although we undertook an exhaustive search, the possibility remains that we were unable to include all relevant outcome evaluations. In addition, our meta-analysis drew on evidence of variable quality, with limited scope for meta-analyses. We were unable to account for heterogeneity between studies where this was present owing to either scarcity of evidence or limitations of our evidence base. In particular, we were unable to undertake network meta-analysis, and most meta-analyses had too few studies to make meta-regression, for example, comparing intervention type on outcomes, meaningful. In our analysis of sexual risk outcomes, which was the one model where we were able to undertake meta-regression, we were unable to explain heterogeneity. Meta-regressions by outcome type to determine differential effectiveness on outcomes within sexual risk would have been uninterpretable owing to the statistical methods used for meta-analysis and owing to multiple studies reporting outcomes across several domains. Probable publication and selective reporting bias across studies meant that several estimates of intervention effectiveness from the included studies could not be included in our meta-analyses; in at least one case, outcomes stated in a trial protocol were not published in the main trial report. Finally, we were unable to locate evidence for some scoped outcomes.

### Conclusion

Our systematic review found that while some evidence exists for the effectiveness of eHealth interventions in addressing sexual risk in MSM, the quality of evidence was poor, as was the quality of the evidence for the range of outcomes considered. eHealth interventions present a potentially powerful avenue for addressing a range of interrelated health challenges; however, more needs to be understood about how interventions work for individual outcomes before progressing a comprehensive intervention.

## Appendix

Multimedia Appendix 1 Additional search details.

Multimedia Appendix 2 Table of included studies.

Multimedia Appendix 3 Study-level risk of bias judgments.

Multimedia Appendix 4 Forest plots for sexually transmitted infection outcomes.

## Abbreviations

GRADE: Grading of Recommendations, Assessment, Development and Evaluations
MSM: men who have sex with men
OR: odds ratio
PROSPERO: Prospective Register of Systematic Reviews
RR: risk ratio
SBS: Safe Behavior and Screening
STI: sexually transmitted infection
