# Supplementary material for: eHealth Interventions to Address HIV and Other Sexually Transmitted Infections, Sexual Risk Behavior, Substance Use, and Mental Ill-health in Men Who Have Sex With Men: Systematic Review and Meta-analysis
Source: JMIR Public Health Surveill. 2022 Apr 6;8(4):e27061. doi: 10.2196/27061 (PMC9021948; doi:10.2196/27061)
Supplement: Multimedia Appendix 1 [file publichealth_v8i4e27061_app1.docx]

**Multimedia Appendix 1.** Search methods to locate studies

**Database searches**

The following databases were searched between October 23^rd^, 2018 and November 26^th^, 2018 and again between April 22^nd^-27^th^, 2020. These databases were selected to retrieve research literature from the fields of health and social sciences. Due to the COVID-19 lockdown in the UK and a lack of access to libraries, the following databases were not included in the update search: OvidSP Health Management Information Consortium (HMIC); ProQuest Applied Social Sciences Index & Abstracts (ASSIA); ProQuest Sociological Abstracts; ProQuest IBSS. We present the search string for OvidSP MEDLINE below, as run in 2018.

1. ProQuest Applied Social Sciences Index & Abstracts (ASSIA) (1987 – current as of October 29^th^, 2018). Due to lack of access, this database search was not updated in April 2020.
2. Campbell Library (complete database as of April 27^th^, 2020)
3. EBSCO CINAHL Plus (complete database as of April 22^nd^, 2020)
4. Wiley Cochrane Library (complete database as of April 27th, 2020)
5. CRD Databases (DARE & NHS EED no longer updated, stopped in 2015. HTA updated as of 26/10/2018). This database had not been updated so was not searched in 2020.
6. EPPI-Centre Database of Health Promotion Research (Bibliomap) (full database as of April 27^th^ 2020)
7. ProQuest Dissertations & Theses Global (1951 – current as of April 27^th^, 2020)
8. OvidSP EconLit (1886 to April 16^th^, 2020)
9. OvidSP Embase (1980 to April 21^st^, 2020)
10. OvidSP Global Health (1910 to 2020 week 15)
11. OvidSP HMIC (Health Management Information Consortium) (1979 to July 2018). Due to lack of access this database search was not updated in April 2020.
12. ProQuest International Bibliography of the Social Sciences (IBSS) (1951 – current as of October 29^th^, 2018). Due to lack of access this database search was not updated in April 2020.
13. Ovid MEDLINE(R) ALL (1946 to April 21^st^, 2020)
14. OvidSP PsycInfo (1806 to April week 2, 2020)
15. Web of Science Science Citation Index Expanded (1970 – present. Data last updated 2020/04/21)
16. Scopus (complete database as of April 22^nd^, 2020)
17. OvidSP Social Policy & Practice (202001)
18. Web of Science Social Sciences Citation Index Expanded (1970 – present. Data last updated 2020/04/21)
19. ProQuest Sociological Abstracts (1952 – current as of October 29^th^, 2018). Due to lack of access this database search was not updated in April 2020.

**Clinical trials registers and grey literature**

We also searched the following clinical trials registers for relevant ongoing and unpublished trials:

1. ClinicalTrials.gov (complete database as of April 27^th^, 2020)
2. World Health Organization International Clinical Trials Registry Platform (ICTRP) (complete database as of November 26^th^, 2018). Due to the COVID-19 pandemic, the ICTRP search functionality was removed. Therefore this search could not be updated.
3. EPPI-Centre Trials Register of Promoting Health Interventions (TRoPHI) (full database as of April 27^th^, 2020).

Search terms were derived from the OvidSP Medline search compiled for database searching. All trial details were examined for their relevance and included if they met our inclusion criteria.

To find other grey literature, the complete OpenGrey database was searched on November 1^st^, 2018 and again on April 27^th^, 2020, using a version of the OvidSP Medline search compiled for database searching. Google was searched in incognito mode, to look for non-governmental organisation and governmental publications on November 21^st^, 2018. Search terms were derived from the OvidSP Medline search compiled for database searching. The first 100 results for each search were examined for their relevance and included if they met our inclusion criteria. The Google search was not updated in 2020.

**Additional search methods**

We also carefully searched reference lists from all studies that meet our inclusion criteria. We contacted subject experts to identify relevant ongoing or completed research. Our protocol specified that we would hand-search journals that published included studies which we found only via reference checking and which are not indexed on databases we have searched, but no journals met this criterion.

**MEDLINE search string**

1. Homosexuality/ (12169)
2. Homosexuality, Male/ (13445)
3. exp "Sexual and Gender Minorities"/ (3131)
4. Bisexuality/ (3695)
5. Transsexualism/ (3421)
6. gender identity/ (17248)
7. Health Services for Transgender Persons/ (92)
8. exp Sex Reassignment Procedures/ (550)
9. homosexual*.ti,ab. (13006)
10. gay.ti,ab. (9392)
11. "men who have sex with men".ti,ab. (9288)
12. MSM.ti,ab. (8276)
13. bisexual*.ti,ab. (7793)
14. gbMSM.ti,ab. (42)
15. (transgender* or trans-gender*).ti,ab. (3923)
16. (transsexual* or trans-sexual*).ti,ab. (2333)
17. (transm#n or trans-men or trans-man).ti,ab. (209)
18. (transwom#n or trans-wom#n).ti,ab. (220)
19. (transfemale? or trans female?).ti,ab. (19)
20. trans people.ti,ab. (82)
21. trans person.ti,ab. (3)
22. tgm.ti,ab. (334)
23. tgw.ti,ab. (180)
24. gender identity.ti,ab. (2272)
25. cross gender.ti,ab. (256)
26. sex reassignment.ti,ab. (516)
27. gender reassignment.ti,ab. (270)
28. gender dysphoria.ti,ab. (646)
29. gender transition.ti,ab. (89)
30. queer.ti,ab. (905)
31. sexual-minorit*.ti,ab. (1751)
32. gender-minorit*.ti,ab. (304)
33. LGBT*.ti,ab. (1350)
34. or/1-33 [MSM] (62357)
35. exp telemedicine/ (23614)
36. ccbt.ti,ab. (144)
37. (ehealth or e-health or electronic health*).ti,ab. (15158)
38. (etherap* or e-therap* or electronic therap*).ti,ab. (426)
39. (eportal or e-portal or electronic portal).ti,ab. (1012)
40. telehealth*.ti,ab. (3111)
41. telemed*.ti,ab. (9034)
42. telemonitor*.ti,ab. (1239)
43. telepsych*.ti,ab. (514)
44. teletherap*.ti,ab. (1309)
45. icbt.ti,ab. (539)
46. (mhealth or m-health).ti,ab. (2109)
47. or/35-46 [GENERAL E-HEALTH] (45055)
48. cell phone/ (7494)
49. wireless technology/ (2864)
50. exp microcomputers/ (19620)
51. cellphone.ti,ab. (178)
52. computer*.ti,ab. (277170)
53. (ipad or i-pad).ti,ab. (1036)
54. (iphone or i-phone).ti,ab. (634)
55. (ipod or i-pod).ti,ab. (287)
56. mobile*.ti,ab. (84502)
57. phone*.ti,ab. (30951)
58. smartphone.ti,ab. (5396)
59. technolog*.ti,ab. (394411)
60. telephon*.ti,ab. (54456)
61. wifi.ti,ab. (281)
62. wireless.ti,ab. (11091)
63. or/48-62 [HARDWARE] (817195)
64. electronic mail/ (2459)
65. text messaging/ (2040)
66. exp videoconferencing/ (1572)
67. exp internet/ (70489)
68. mobile applications/ (3439)
69. virtual reality/ (502)
70. android.ti,ab. (1874)
71. (app or apps).ti,ab. (22044)
72. blog*.ti,ab. (1537)
73. cyber*.ti,ab. (5586)
74. (email* or e-mail*).ti,ab. (13513)
75. facebook.ti,ab. (2501)
76. instagram.ti,ab. (215)
77. instant messag*.ti,ab. (247)
78. internet*.ti,ab. (43734)
79. media-based.ti,ab. (796)
80. media-deliver*.ti,ab. (51)
81. messag* service?.ti,ab. (1044)
82. (multimedia or multi-media).ti,ab. (4808)
83. new-media.ti,ab. (621)
84. (online* or on-line*).ti,ab. (114701)
85. podcast*.ti,ab. (618)
86. reddit.ti,ab. (56)
87. social network* site*.ti,ab. (944)
88. sms.ti,ab. (4906)
89. snapchat.ti,ab. (31)
90. social-medi*.ti,ab. (9271)
91. software.ti,ab. (138893)
92. telecomm*.ti,ab. (3877)
93. text-messag*.ti,ab. (3005)
94. texting.ti,ab. (667)
95. twitter.ti,ab. (2077)
96. video-based.ti,ab. (1897)
97. virtual*.ti,ab. (113968)
98. vlog*.ti,ab. (29)
99. web*.ti,ab. (125844)
100. www.ti,ab. (1454)
101. youtube.ti,ab. (1273)
102. or/64-101 [SOFTWARE OR MEDIA] (565472)
103. "Cell Phone Use"/ (56)
104. 47 or 63 or 102 or 103 [ALL EHEALTH] (1310855)
105. 34 and 104 [MSM AND EHEALTH] (5016)
106. limit 105 to yr="1995 -Current" (4709)
107. remove duplicates from 106 (4701)
