# Supplementary material for: eHealth Interventions to Address HIV and Other Sexually Transmitted Infections, Sexual Risk Behavior, Substance Use, and Mental Ill-health in Men Who Have Sex With Men: Systematic Review and Meta-analysis
Source: JMIR Public Health Surveill. 2022 Apr 6;8(4):e27061. doi: 10.2196/27061 (PMC9021948; doi:10.2196/27061)
Supplement: Multimedia Appendix 2 [file publichealth_v8i4e27061_app2.docx]

**Multimedia Appendix 2.** Table of included studies

| Trial | Sample details | Intervention | Outcomes |
| --- | --- | --- | --- |
| Bauermeister et al (2019) [26] | Location – country (region): United States  Target population: Single, young, HIV-negative MSM who report condomless anal intercourse (CAI) with partners met online  Sampling: Recruitment conducted via ads on online social and sexual networking sites. Eligible participants were single, cisgender males aged 18-24 years of negative or unknown HIV status who report unprotected anal intercourse with male partner met online.  Sample size (overall response rate), baseline: N=180 enrolled and randomized  Sexuality:  Overall: 88.3% gay, 7.8% bisexual, 3.9% queer  Included in analysis: 89% gay, 7% bisexual, 4% queer  Gender identity: Not stated; eligible participants were MSM  Ethnicity: Overall: 67.2% White, 16.1% Multiracial, 10.0% Black, 5.6% Asian, 0.6% Middle Eastern, 0.6% Native American  Included in analysis: 67% White, 16% Multiracial, 10% Black, 5% Asian, 1% Middle Eastern, 1% Native American  Socio-economic status: Overall: 2.8% some high school, 10.6% high school diploma or GED equivalent, 7.8% technical or associate degree, 40.0% some college, 29.4% graduated college, 9.5% reported attending graduate school  Included in analysis: Not stated  Age: Overall: mean 21.67 years (SD=1.81)  Included in analysis: mean 21.5 (SD=1.82)  Sample size (overall response rate), follow-up:  91% completed at least 1 follow-up assessment  30-day: N=143 (79%)  60-day: N=150 (83%)  90-day: 147 (82%)  Included in analysis: N=155  Included in sexual behavior outcome analyses: N=123  Intervention: N=95  Control: N=28 | Description: Modular HIV prevention intervention  Technology: Internet  Timing and duration: 6 sessions, which could be accessed for 90 days.  Target population: Young adult MSM  Theoretical framework: Guided by a dual processing cognitive-emotional decision-making framework myDEx targets both cognitive factors (e.g., knowledge, skills and self-reflection) and emotional factors including limerence. Each session includes activities and videos to build HIV risk reduction skills and promote self-reflection.  Content: This module-based comprehensive sex education intervention aimed to improve psychological well-being and reduce HIV risk by targeting condom use; HIV/STI testing; unprotected anal intercourse; PrEP; and alcohol/drug use before sex. Content within each session was organized into three levels: a core message, deeper discussion of relevant topics and an activity. Content used story-telling, case scenarios, motivational interviewing strategies, graphics and videos, and it was tailored to the user via personalization, content matching and feedback to maximize persuasiveness and relevance. Interactive activities included role-play scenarios, a diary, quizzes and opportunities to develop dating strategies.  Control: Information-only attention-control contained six sessions matching myDEx design. Content mirrored the US Center for Disease Control and Prevention’s HIV Risk Reduction Tool. | Condomless receptive anal intercourse during 3-month trial period  *X _(1)_*^2^ = 4.40, *p*=0.04; OR=0.43, 95%CI (0.20, 0.94)  Condomless receptive anal intercourse with serodiscordant or serounknown partners not known to be on PrEP or virally suppressed  *X_(1)_*^2^=2.18, *p*=0.14, OR=0.44, 95%CI (0.15, 1.31)  Insertive CAI  *X_(1)_*^2^=1.19, *p*=0.27, OR=0.64, 95%CI (0.28, 1.44)  Insertive CAI w/serodiscordant or serounknown partners not known to be on PrEP or virally suppressed  *X_(1)_*^2^=1.86, *p*=0.16, OR=0.49, 95% CI (0.17, 1.33) |
| Bowen et al (2008) [39] | Location – country (region): United States (rural)  Target population: Rural MSM  Sampling: Banner ads nationwide. Eligible participants were rural MSM aged 18 or older reporting sex with a man in the prior 12 months  Sample size (overall response rate), baseline:  N=425 completed pre-test and were randomized (69% of those eligible)  Sexuality:  Completers (completing all intervention and questionnaire components): 84.4% gay, 15.3% bisexual, 0.3% heterosexual  Gender identity: Not stated; eligible participants were MSM  Ethnicity: Completers (completing all intervention and questionnaire components): 77.2% non-Hispanic white, 8.8% Hispanic, 13.9% Asian/Asian Pacific Islander, African American, Native American, or other  Socio-economic status:  Completers (completing all intervention and questionnaire components)  Education  19.7% high school or less; 80.3% some college or more  Employment  55.7% full-time, 17.9% part-time, 26.5% unemployed/retired  Income  36.7% <$15,000; 25.9% $15,000-$24,999; 27.9% $25,000-$49,999; 9.5% >=$50,000  Age:  Completers (completing all intervention and questionnaire components)  46.8% ages 18-24; 31.1% ages 25-34; 15% ages 35-44; 7.2% ages 45-80  Sample size (overall response rate), follow-up: Completers – completed all intervention and questionnaire components: N=294 (69% of those randomized) | Description: Online modular HIV risk reduction intervention for MSM in rural areas  Technology: Internet  Timing and duration: 3 modules, each comprising 2 20-minute interactive sessions. There had to be at least 48 hours between sessions, meaning the minimum time to complete the intervention was 10 days. Results found that participants took an average of 19.39 days to complete the entire intervention.    Target population: Sexually active, internet-using MSM in rural areas  Theoretical framework: WRAPP was informed by social cognitive theory and the IMB model. The ‘knowledge’ module aimed to increase HIV knowledge. The ‘partner’ module aimed to increase motivation (comprising outcome expectancies for risk reduction and willingness to reduce HIV risk behaviors). The ‘contexts of risk’ module aimed to develop behavioral skills. In turn, knowledge, motivation and behavioral skills were theorized to increase sexual self-efficacy (comprising mechanical self-efficacy – such as self-efficacy for correct condom use – and self-efficacy to refuse condomless anal intercourse), theorized as a direct precursor of behavior change.  Content: Online modular HIV risk reduction intervention. Module content included information tailored for rural MSM and was presented as conversations between gay men. Dialogue was interspersed with interactive activities and graphics. The first module featured a conversation between an HIV-positive gay man who represented an ‘expert’ and an ‘inexperienced’ HIV-negative gay man who had recently had a high-risk sexual encounter, primarily addressing HIV prevention during sex and living with HIV. It featured links to websites with further information.  The second module featured a conversation between 5 gay male friends with one representing the user and aimed to increase motivation, and a third module targeting behavioral skills in a similar format was introduced. Both allowed users to print a summary of their responses to interactive components. The ‘motivation’ module helped users identify reasons for not using condoms and ways to address these to support the user’s pursuit of their life goals. The ‘behavior’ module addressed approaches for reducing sexual risk with partners met online or in a bar.  Control: Not applicable | No specific comparative estimates for differences between groups on condom use index (% of anal intercourse partners with whom condoms were used) but no differences significant between arms |
| Carpenter et al (2010) [27] | Location – country (region): United States  Target population: Young MSM who are having unsafe sex, including minority MSM  Sampling: Recruited via banner advertisements on same-sex community websites and profiles of the study on three other websites. Eligible participants were US men aged 18-39 years with Internet access and a negative or unknown HIV status who had engaged in unprotected oral or anal intercourse with a man in the last 3 months  Sample size (overall response rate), baseline: Overall: N=199 completed baseline measures and were randomized  Intervention: N=99  Control: N=100  Sexuality: Not stated; only MSM eligible  Gender identity: Not stated; only MSM eligible  Ethnicity: 80% White, 15% Hispanic/Latino, 7% Native American, 6% African American, 5% Asian American, 1% Hawaiian/Pacific Islander, 3% Other  Socio-economic status:  Annual income  16% under $10,000/year; 20% $10,000-$20,000/year; 36% $21,000-$40,000/year; 21% $41,000-$60,000/year; 8% $61,000 or over per year  Highest grade (school year) completed  3% less than 12^th^ grade or GED, 13% 12^th^ grade or GED, 12% 1 year college/tech school, 13% 2 years college/tech school, 59% >2 years college/tech schools  Age: Not stated  Sample size (overall response rate), follow-up  Overall: N=112 completed follow-up measures (56% of those randomized; 78% of those who completed the intervention and were sent a link to the follow-up survey)  Intervention: N=59 (60% of those randomized to intervention, 80% of those receiving survey link)  Control: N=53 (53% of those randomized to control, 77% of those receiving survey link) | Description: Multi-media, modular HIV/STI intervention  Technology: Internet  Timing and duration: 7 brief sequential modules completed within 1 week. Authors’ description suggests intervention took approximately 1.5 hours. Participants could return to view intervention content during the follow-up period.  Target population: Young MSM, including minority MSM  Theoretical framework: Based on the IMB Model, the intervention aimed to reduce risk of HIV and other STIs by addressing information, motivation and behavioral skills. The information component aimed to increase knowledge of risk factors. Intervention activities assessed readiness to change and incorporated ‘stage-based’ and (informed by motivational interviewing approaches) decisional balance exercises to increase motivation. Informed by motivational interviewing, the intervention also assessed HIV risk factors to inform targeted feedback, and identified perceived barriers to change in order to increase self-efficacy.  Content: This website-based intervention aimed to reduce HIV/STIs via modules addressing information about risk factors, skills (e.g., partner communication) and motivation. Multimedia content included didactic materials, quizzes, interactive exercises and audio from simulated peers. The approach was non-judgmental and emphasized both responsibility and freedom of choice. User assessments informed motivational exercises tailored to the user’s readiness to change as well as tailored feedback.  Control: Stress reduction training Programme originally developed for the general population and customized for young MSM by substituting representative photographs. | With partner of positive/unknown serostatus  Time effect: F 3.25; df 597; p=0.009; n2 0.144  Group x time: F 4.13; df 597; p=0.002; n2 0.175  Unprotected anal intercourse  Time effect: F 7.59; df 1,101; p=0.007; n2 0.070  Group x time: F 7.59; df 1,101; p=0.007; n2 0.070  Unprotected insertive anal intercourse  Time effect: F 3.37; df 1,101; p=0.069; n2 0.032  Group x time: F 7.24; df 1,101; p=0.008; n2 0.067  Unprotected receptive anal intercourse  Time effect: F 4.79; df 1,101; p=0.031; n2 0.045  Group x time: F 1.35; df 1,101; p=0.248; n2 0.013  Unprotected insertive oral intercourse  Time effect: F 13.88; df 1,101; p=<0.001; n2 0.121  Group x time: F 7.45; df 1,101; p=0.007; n2 0.069  Unprotected receptive oral intercourse  Time effect: F 13.24; df 1,101; p=<0.001; n2 0.116  Group x time: F 8.45; df 1,101; p=0.004; n2 0.077 |
| Cheng et al (2019) [28] | Location – country (region): China  Target population: MSM  Sampling: Recruited via advertisements on popular gay website. Eligible participants were male Internet users aged 18 years and older reporting sex with men in prior 6 months; excluded those who participated in an HIV intervention before.  Sample size (overall response rate), baseline: Overall: N=1,100 completed baseline survey and were randomized  Intervention: N=550  Control: N=550  Sexuality: Overall: 78% homosexual  Control: 78% homosexual, 22% bisexual/heterosexual/other  Intervention: 78% homosexual, 22% bisexual/heterosexual/other  Gender identity: Not stated; eligible participants were MSM  Ethnicity:  Control: 98% Han, 2% minority  Intervention: 97% Han, 4% minority  Socio-economic status:  Education  Overall: 80% college or above  Control: 4% junior high school or below, 19% senior high school, 78% college or above  Intervention: 3% junior high school or below, 15% senior high school, 82% college or above  Annual Income  Control: 9% no income, 37% less than $5,351; 36% $5,351-$12,485; 18% $12,485 or above  Intervention: 7% no income, 29% less than $5,351; 44% $5,351-$12,485; 20% $12,485 or above  Age:  Overall: 62% were aged 21-30 years  Control: Age in years: 6% aged ≤20, 61% aged 21-30, 27% aged 31-40, 6% aged ≥41  Intervention: Age in years: 4% ≤20, 64% aged 21-30, 25% aged 31-40, 7% aged ≥41  Sample size (overall response rate), follow-up: N=986 (90%) completed post-survey  Intervention: N=501 (91%) completed post-survey  Control: N=485 (88%) completed post-survey | Description: Two-part HIV prevention intervention delivered on popular gay website  Technology: Online  Timing and duration: Part I delivered immediately after completing baseline survey. Following completion of Part I, Part II delivered in 3 parts each delivered weekly.  Target population: MSM  Theoretical framework: Informed by the theory of planned behavior, the intervention targeted attitudes, subjective norms, perceived control and behavioral intention which are posited as key determinants of health behaviors. It aimed to increase knowledge and reduce misconceptions. Part I aimed to engage participants and increase HIV risk perceptions by presenting realistic scenarios and to increase awareness of community norms by presenting peer attitudes towards behavioral decisions. Part II addressed basic HIV/AIDS knowledge and transmission; presented information about the HIV epidemic among MSM, aiming to increase HIV risk perception and reduce sexual risk behaviors; and addressed misconceptions about sexual behaviors.  Content: 2-part interactive HIV prevention intervention delivered via popular gay website in China. Part I comprised realistic interactive scenarios addressing sexual behavior (CAI, condom breakage, encountering sex partner in a pub and commercial sexual encounter) and HIV testing, and it presented peers’ attitudes towards behavioral decisions. Part II presented visually appealing HIV information tailored for MSM addressing HIV/AIDS basic knowledge and transmission, local epidemic data among MSM and sexual behaviors.  Control: Standard HIV referral service, also provided to intervention participants: recommendation for HIV test at local clinic | Condomless anal intercourse in prior 3 months  Completed records (N=986): Estimated risk difference (difference in proportions)=9.3% (95% CI=1.1, 17.5)  Multiple imputation ITT (N=1,100): Estimated risk difference (difference in proportions)=8.9% (95% CI= 1.2, 16.6) |
| Chiou et al (2020) [29] | Location – country (region): Taiwan  Target population: HIV-negative MSM  Sampling: Recruitment was conducted via social media platforms and respondent-driven sampling. Eligible participants were HIV-negative MSM aged 20 years and older who had not used an HIV prevention/treatment app in the past year.  Sample size (overall response rate), baseline:  Overall: N=300  Intervention: N=150  Control: N=150  Sexuality: Not stated; eligible participants were MSM  Gender identity: Not stated; eligible participants were MSM  Ethnicity: Not stated  Socio-economic status:  Education  Overall: 65% college or university  Intervention: 64% college or university, 21% above university, 15% high school or less  Control: 66% college or university, 20% above university, 14% high school or less  Employment  Overall: 78% employed  Intervention: 77% employed, 21% student, 2% unemployed  Control: 79% employed, 19% student, 3% employed  Age:  Overall: Mean 27 years (SD=5.6)  Intervention: Mean 27.4 years (SD=6.3)  Control: Mean 27 years (SD=4.7)  Sample size (overall response rate), follow-up:  Overall: N=265  Intervention: N=130  Control: N=135 | Description: HIV prevention app targeting sexual risk behaviors and recreational drug use  Technology: Smartphone app  Timing and duration: App was used for 6 months; quiz and prize activity related to HIV testing, safe sex and drug use conducted every 3 weeks  Target population: MSM  Theoretical framework: Drew on the IMB Model, which posits that information, behavioral motivation and skills influence HIV prevention behavior. App content provided information which aimed to increase knowledge. Survey measures suggest the intervention also targeted motivation (comprised of attitudes towards reducing risky sexual behavior and recreational drug use, and intention to change these behaviors) and behavioral skills for HIV prevention (including partner communication, negotiating safe sex, drug and unsafe sex refusal skills and correct condom use).  Content: App with 5 features: (1) log to record sexual behavior and recreational drug use which can output tables/figures showing changes over time, and links to PrEP resources; (2) information on HIV/STIs; safe sex strategies including partner communication; recreational drug use including alternative strategies to enhance arousal before sex; and PrEP; (3) recommendations, links and a log to promote and record testing; (4) search, messaging and message board to interact with other users; and (5) presentation of most popular users, message boards and testing locations  Control: No programming offered to control arm | Condom use during anal intercourse: *B*=20.7 (SE=0.058), *t*=3.536, *p*=0.001  Recreational drug use: *B*= -1.19 (SE=0.204), *t*= -5.850, *p*<0.001  HIV-positive rate: IRR=1.56 (95%CI=0.258, 9.557), *p*=0.842  Syphilis positivity rate: IRR=1.39 (95%CI=0.307, 6.366), *p*=0.664 |
| Christensen et al (2013) [30] | Location – country (region): United States  Target population: Young adult MSM  Sampling: Recruited via clickable banner ads on websites used by MSM. Eligible participants were Black/African American, Hispanic/Latino or White/Caucasian MSM aged 18-24 years, reported a prior HIV-negative test result and reported CAI with a non-primary male partner in prior 3 months.  Sample size (overall response rate), baseline:  Overall: N=935 allocated to study arm, completed baseline (46% of those randomized)  Intervention: N=444 received allocated intervention and completed baseline measures (35% of those randomized)  Control: N=491 received allocated control and completed baseline measures (67% of those randomized)  Sexuality:  Intervention: 74.5% gay/homosexual, 14.7% bisexual, 10.4% other  Control: 76.6% gay/homosexual, 11.4% bisexual, 11.6% other  Gender identity:  Not stated; eligible participants were MSM  Ethnicity:  Intervention: 76.1% White/Caucasian, 12.4% Latino/Hispanic, 11.5% Black/African American  Control: 71.1% White/Caucasian, 15.5% Latino/Hispanic, 13.4% Black/African American  Socio-economic status:  Education  Intervention: 83.6% have at least some postsecondary education  Control: 80.2% have at least some postsecondary education  Residence  Intervention: 12.8% live in rural geographic area  Control: 13.4% live in rural geographic area  Age:  Intervention: mean 21.3 years (SD=1.8)  Control: mean 21.3 years (SD=1.7)  Sample size (overall response rate), follow-up:  Overall: N=628 (67% of those completing baseline)  Intervention: N=294 (66%)  Control: N=334 (68%) | Description: Animated game for HIV prevention, simulating situations typically confronted by young adult MSM on first dates or ‘hook-ups’  Technology: Computer download  Timing and duration: 30 minutes  Target population: Young adult MSM  Theoretical framework: Via multiple theorized pathways, SOLVE aimed to decrease unprotected anal intercourse thereby reducing HIV risk. Informed by the notion that shame due to ‘sexual stigma’ can contribute to HIV risk behaviors, SOLVE simulated shame-inducing situations; promoted conscious acknowledgement and normalization of the user’s desires; and role-modelled positive attitudes towards one’s self as well as comfort with the user’s sexuality and desires. The authors’ description suggested this aimed to decrease shame by normalizing MSMs’ desires, increasing self-worth and self-acceptance and reducing isolation and feelings of inferiority. Drawing on neuroscience research suggesting that emotions play a critical role in decision-making, SOLVE aimed to increase self-awareness of goals, emotions and barriers to safer sex; promote recognition of the consequences of the user’s desires; interrupt affect-based decision-making and increase self-regulation. Other components aimed to increase HIV knowledge and hone HIV risk-reduction skills and strategies.  Content: SOLVE aimed to decrease unprotected anal intercourse thereby reducing HIV risk. In this 3-D animated game, the user took the role of a customizable avatar and made decisions which affected the narrative in simulated settings presenting risky situations and barriers to safer sex that young adult MSM typically confront on first dates or ‘hook-ups.’ The intervention simulated shame-inducing situations, and the avatar and other guide characters modelled acceptance and normalization of the user’s desires. At decision points these characters used an ‘ICAP’ involving: “(I) interrupting automatic risky choices, (C) challenging those choices with persuasive messages, (A) acknowledging, accepting and sharing MSM’s emotions/motives (e.g., desires for men) and (P) providing a way and skills for MSM to be safe.”(p.2)  Control: Waitlist control | Shame change predicted CAI change (N=921):  B=0.73, SE=0.36, 95% bias-corrected CI=0.03, 1.45  Indirect effect on CAI change  Point estimate = -0.10, 95% bias-corrected CI = -0.01, -0.23 |
| Davidovich et al (2006) [31] | Location – country (region): The Netherlands  Target population: Single males open to a steady relationship with a man in the future  Sampling: Eligible participants were male, HIV-negative or of unknown serostatus, single, and open to a steady relationship with a man in the future. They were recruited via websites popular among gay men in the Netherlands.  Sample size (overall response rate), baseline:  Overall: N=1,013 randomized following baseline questions  Intervention group  Non-tailored: N=340  Tailored: N=340  Control group: N=333  Sexuality: 63% exclusively attracted to men, 18% primarily attracted to men, 17% equally attracted to men and women, and 2% primarily attracted to women  Gender identity: All-male sample  Ethnicity: 21% non-Dutch  Socio-economic status: 53% university-level or equivalent  Age: Mean 33 years (SD=11.1)  Sample size (overall response rate), follow-up: Overall : N=375 (37.0%)  Represents 56% of the sample that provided their email address and could therefore be contacted at follow-up  Of these, N=130 (35%) had a new steady partner by follow-up and could be included in the follow-up analysis. NB, intervention and control Ns for this subgroup not specified.  Intervention group  Non-tailored: N=107 (31%)  Tailored: N=128 (38%)  Control group: N=140 (42%) | Description: HIV prevention intervention promoting negotiated safety (i.e., unprotected anal intercourse between steady partners who are both HIV-negative)  Technology: Internet  Timing and duration: Users spent an estimated mean of 30 minutes in the non-tailored version and 10-30 minutes in the tailored version  Target population: Single gay men    Theoretical framework: Informed by the IMB Model, modules addressed information, motivation and behavioral skills; the motivation component was further informed by the theory of planned behavior and the health belief model. Information modules aimed to increase response efficacy for practicing negotiated safety (comprising knowledge and beliefs). Motivation modules aimed to correct faulty beliefs in order to shape attitudes and aimed to increase perceptions of HIV testing benefits and sense of vulnerability. Attitudes were theorized to increase condom use intentions; and attitudes, sense of vulnerability and perceived benefits of HIV testing were theorized to increase intentions to practice negotiated safety.  Content: There were two versions of the intervention: A non-tailored version delivered all modules, and a tailored version delivered general content considered relevant for all users in addition to selected modules considered relevant based on a baseline questionnaire. Information modules addressed how to practice negotiated safety; motivation modules addressed HIV transmission risk via steady partners, HIV testing and sexual agreements and stressed the consequences of HIV infection; and skills modules taught skills for negotiated safety.  Control: Waitlist control | Multinomial logistic regression  Negotiated safety vs. risky CAI, OR (95% CI)  Tailored: 10.50 (1.19, 92.72)  Non-tailored : 1.62 (0.14, 19.07)  Condom vs. risky CAI, OR (95% CI)  Tailored arm: 1.66 (0.68, 4.02)  Non-tailored arm: 0.55 (0.22, 1.37) |
| Hirshfield et al (2019) [32] | Location – country (region): United States  Target population: MSM living with HIV  Sampling: Recruitment was conducted via ads on social and sexual networking websites, online bulletin boards, GPS-based apps and an email blast to members of online dating site for people living with HIV. Eligible participants were MSM identifying as Black, White or Hispanic; were living with HIV, with a detectable viral load or suboptimal ART adherence; and reported CAI with known or unknown serodiscordant male partners  Sample size (overall response rate), baseline:  Overall: N=830 eligible and randomized  Intervention: N=413  Control: N=417  Sexuality: Not stated; eligible participants were gay, bisexual and other MSM  Gender identity: Not stated; eligible participants were assigned male at birth and identified as male or genderqueer    Ethnicity:  Intervention: 50% White, 27% Black, 23% Hispanic  Control: 50% White, 26% Black, 24% Hispanic  Socio-economic status:  Education  Intervention: 12% high school or less, 40% some college or enrolled, 48% college degree or more  Control: 11% high school or less, 46% some college or enrolled, 43% college degree or more  Annual income  Intervention: 17% less than $10,000; 18% $10,000-$19,999; 26% $20,000-$39,999; 16% $40,000-$59,999; 13% 60,000-$99,999; 9% $100,000 or more  Control: 17% less than $10,000; 19% $10,000-$19,999; 27% $20,000-$39,999; 15% $40,000-$59,999; 16% 60,000-$99,999; 7% $100,000 or more  Region  Intervention: 4% rural, 9% small town, 4% suburb of a smaller urban area, 8% smaller urban area, 20% suburb of a big city, 56% big city  Control: 4% rural, 5% small town, 5% suburb of a smaller urban area, 11% smaller urban area, 22% suburb of a big city, 54% big city  Age:  Intervention: Age in years: 8% aged 18-24, 19% aged 25-29, 32% aged 30-39, 24% aged 40-49, 17% aged 50+  Control: Age in years: 8% aged 18-24, 16% aged 25-29, 30% aged 30-39, 28% aged 40-49, 19% aged 50+  Sample size (overall response rate), follow-up:  Overall  3-month survey: N=667 (80%)  12-month survey: N=606 (73%)  Intervention  3-month survey: N=327 (79%)  12-month survey: N=292 (71%)  Control  3-month survey: N=340 (81%)  12-month survey: N=314 (75%) | Description: Video-based intervention to prevent onward HIV transmission  Technology: Internet  Timing and duration: 6 videos, delivered weekly for 3 months, and 4 booster videos delivered weekly starting at 6 months. Full intervention was delivered over a 1-year period.  Target population: MSM living with HIV  Theoretical framework: Informed by social cognitive theory and social learning theory, Sex Positive! aimed to prevent onward HIV transmission among MSM living with HIV. The dramatic series sought to optimize engagement by featuring stories and characters with which target users would identify. Informed by social learning theory, it used modelling to demonstrate risk reduction and health behaviors including HIV disclosure and discussions about safer sex. Content aimed to promote critical thinking about HIV disclosure, medication adherence, viral suppression, sex under the influence of drugs or alcohol and sero-discordant condomless anal intercourse. Authors’ narrative and the constructs assessed in user surveys suggested critical thinking was theorized to promote self-efficacy for safer sex and for HIV status disclosure to partners; promote perceived personal and partner responsibility for preventing HIV transmission; and shape outcome expectancies for condoms, anal intercourse and HIV disclosure. Modelling of self-regulation aimed to improve skills for regulating sexual compulsivity. These mediators were theorized to influence HIV treatment adherence, mental health, substance use, sexual behavior and interpersonal violence outcomes. Four follow-up booster videos aimed to help sustain impact over time.  Content: The intervention’s dramatic video series “Just a Guy” followed “Guy,” a gay man living with HIV in Brooklyn, New York. The intervention used modelling to demonstrate risk reduction and health behaviors, including HIV disclosure and discussions about safer sex. Four follow-up booster videos aimed to help sustain the intervention’s impact over time.  Control: Attention-control video arm consisted of 10 videos on healthy living, of comparable length to intervention videos (2-4 minutes) and delivered on the same schedule: weekly for 6 weeks, then weekly for 4 weeks following the 6-month assessment. | Change in known serodiscordant CAI partners between baseline and 3-months (N=344)  One or more fewer partners: Risk difference= -3.7, Risk ratio=0.84 (95% CI=0.55, 1.26)  No change in partners: Risk difference=9.8, Risk ratio=1.19 (95% CI=0.99, 1.44)  One or more additional partners: Risk difference= -6.1, Risk ratio=0.77 (95% CI=0.52, 1.13)  Unadjusted linear regression: R2=0.000, B= -0.006, SE B=0.094, β= -0.003, 95% CI= -0.189, 0.178, p=0.95  Adjusted linear regression: R2=0.232, B= 0.005, SE B=0.088, β= 0.003, 95% CI= -0.168, 0.178, p=0.96  Change in known serodiscordant CAI partners between baseline and 12-months (N=281)  One or more fewer partners: Risk difference= -7.4, Risk ratio=0.76 (95% CI=0.51, 1.12)  No change in partners: Risk difference= -0.8, Risk ratio=0.98 (95% CI=0.76, 1.27)  One or more additional partners: Risk difference=8.3, Risk ratio=1.36 (95% CI=0.92, 2.01)  Unadjusted linear regression: R2=0.007, B= -0.156, SE B=0.115, β= -0.081, 95% CI= -0.382, 0.070, p=0.17  Adjusted linear regression: R2=0.374, B= -0.141, SE B=0.097, β= -0.073, 95% CI= -0.332, 0.051, p=0.15  Change in unknown serodiscordant CAI partners between baseline and 3-months (N=376)  One or more fewer partners: Risk difference=6.0, Risk ratio=1.20 (95% CI=0.90, 1.60)  No change in partners: Risk difference=4.0, Risk ratio=1.09 (95% CI=0.88, 1.35)  One or more additional partners: Risk difference= -10.0, Risk ratio=0.60 (95% CI=0.39, 0.92)  Unadjusted linear regression: R2=0.006, B=0.159, SE B=0.106, β=0.077, 95% CI= -0.049, 0.366, p=0.13  Adjusted linear regression: R2=0.319, B=0.146, SE B=0.099, β=0.071, 95% CI= -0.050, 0.341, p=0.14  Change in unknown serodiscordant CAI partners between baseline and 12-months (N=299)  One or more fewer partners: Risk difference= -3.4, Risk ratio=0.91 (95% CI=0.68, 1.23)  No change in partners: Risk difference= -2.1, Risk ratio=0.95 (95% CI=0.72, 1.25)  One or more additional partners: Risk difference= 5.5, Risk ratio=1.27 (95% CI=0.84, 1.93)  Unadjusted linear regression: R2=0.006, B= -0.162, SE B=0.122, β= -0.076, 95% CI= -0.402, 0.079, p=0.19  Adjusted linear regression: R2=0.334, B= -0.177, SE B=0.113, β= -0.084, 95% CI= -0.399, 0.045, p=0.12 |
| Milam et al (2016) [33] | Location – country (region): United States (southern California)  Target population: HIV-positive MSM reporting unprotected sex or STIs  Sampling: Eligible participants were HIV-positive MSM ages 18+ with risk of HIV transmission. Participants were recruited from 3 HIV clinics.  Sample size (overall response rate), baseline: Overall: N=181 were randomized. mITT sample was 179 (2 who were randomized did not complete baseline visit).  Intervention: N=90  Control: N=89  Sexuality: Not stated, but eligible participants were MSM  Gender identity: Not stated, but eligible participants were MSM  Ethnicity  Intervention: 36% White, 31% Black, 31% Hispanic, 3% Other  Control: 30% White, 30% Black, 32% Hispanic, 8% Other  Socio-economic status:  Income  Intervention: 22% have income ≥ $2000/month  Control: 24% have income ≥ $2000/month  Education  Intervention: 77% education more than high school  Control: 75% education more than high school  Age  Intervention: 44.6 years  Control: 42.7 years  Sample size (overall response rate), follow-up: mITT sample (randomized and completed baseline visit)  N=179 (99% of those randomized)  Intervention: N=90  Control: N=89 | Description: Web-based safer sex intervention tailored to user’s risk level, behavior and intentions  Technology: Internet  Timing and duration: Brief intervention provided monthly for 1 year  Target population: HIV-positive MSM  Theoretical framework: This intervention aimed to reduce HIV/STI transmission by targeting condom use, disclosure to sex partners, ART initiation and reduced use of drugs and alcohol. Informed by social cognitive theory and the trans-theoretical model, users were directed to web pages tailored to their risk level, behavior and intent related to the targeted behavior change.  Content: This intervention aimed to reduce HIV/STI transmission by HIV-positive MSM by targeting: condom use, disclosure to sex partners, ART initiation and reduced use of drugs and alcohol. Based on their responses to monthly sexual behavior surveys, the user was directed to static web pages tailored to their risk of transmission. Tailored messaging took into account the user’s current behavior and intent related to the targeted behavior change.  Control: Brief monthly sexual behavior survey accessed via computer for 1 year | Incident STI event over 12-month period  Primary analysis, mITT sample: OR=1.35 (95% CI=0.68, 2.70), p=0.38 |
| Mustanski et al (2013) [34] | Location – country (region): United States (Chicago, Illinois)  Target population: Ethnically/racially diverse young MSM receiving an HIV-negative test result at a clinic.  Sampling: Recruited face-to-face by clinic staff upon receipt of negative HIV test result. Eligible participants were sexually active MSM ages 18-24, were not in an exclusive relationship lasting longer than 12 months and reported internet use.  Sample size (overall response rate), baseline:  N=102 consented, completed baseline assessment and were randomized (84% of those eligible)  Intervention: N=50  Control: N=52  Sexuality: Intervention: 78% gay/homosexual, 22% bisexual/other  Control: 87% gay/homosexual, 14% bisexual/other  Gender identity: Not stated; eligible participants had male birth sex and gender identity  Ethnicity:  Intervention: 46% White-Latino, 24% White-Non-Latino, 14% African American, 16% Other  Control: 46% White-Latino, 27% White-Non-Latino, 12% African American, 15% Other  Socio-economic status:  Employment  Intervention: 56% employed  Control: 73% employed  Education  Intervention: 24% some high school or graduated high school; 76% some college or graduated college  Control: 21% some high school or graduated high school; 79% some college or graduated college  Age:  Intervention: mean of 21.62 years (SD=1.97)  Control: mean of 21.04 years (SD=1.69)  Sample size (overall response rate), follow-up:  Overall: N=90 (88% of those completing baseline)  Intervention: N=41 (80%)  Control: N=49 (94%) | Description: Multi-module, interactive HIV prevention intervention for young MSM from all racial and ethnic groups.  Technology: Internet  Timing and duration: Seven modules totaling approximately two hours, completed across three sessions done at least 24 hours apart. A booster module took place at 6 weeks.  Target population: Ethnically and racially diverse young MSM  Theoretical framework: Informed by the IMB Model, intervention activities were theorized to engender knowledge, motivation and behavioral skills as well as self-efficacy. Reflection was theorized to impact behavioral intentions, an examination of safer sex practices, perceived social norms and a sense of vulnerability which, along with the identification of sources of support, were theorized to contribute to motivation. Participants were recruited following a negative HIV test, a time when they were believed to be particularly receptive to HIV prevention efforts.  Content: Online modules were based on situations and settings relevant to young MSM and used a variety of media and methods such as video, animation and games. The modules addressed, among other topics, condom use; triggers for unprotected sex; obtaining support; communication; the effects of mood, drug and alcohol abuse and sexual arousal; power dynamics in relationships; and the limits of serosorting. In the last module users developed an HIV/STI prevention plan. Goals were suggested tailored to users’ baseline risks. In the booster users revisited goals, received tailored feedback to trouble-shoot obstacles and set new or reaffirmed existing goals.  Control: Online didactic (information-only), non-interactive, non-tailored HIV knowledge information. Control was matched to the intervention in the number of modules and the requirement to participate in them over three sessions. Total time to complete the sessions was not matched to the intervention. | Unprotected anal intercourse acts (N=63)  Rate ratio=0.56, p=0.04  Condom errors  d=0.19, p=0.56  Condom failures  d=0.22, p=0.30 |
| Mustanski et al (2018) [35] | Location – country (region): United States  Target population: Young MSM  Sampling: Recruitment was via community-based HIV testing organizations, local health departments, street outreach and local and national advertising. Eligible participants were sexually active MSM aged 18-29 years who were not in a monogamous relationship of >6 months and tested HIV-negative at screening    Sample size (overall response rate), baseline: N=901 completed baseline assessment and STI testing and were randomized (59% of those eligible)  Intervention: N=445  Control: N=456  Sexuality:  Intervention: 86.5% gay, 11.9% bisexual, 1.6% straight/other  Control: 86.0% gay, 11.2% bisexual, 2.9% straight/other  Gender identity: Not stated; eligible participants were assigned male at birth and identified as male    Ethnicity:  Intervention: 37% White, 24% Black, 30% Hispanic/Latino, 9% Other  Control: 36% White, 25% Black, 27% Hispanic/Latino, 12% Other  Socio-economic status  Intervention: 16% high school or less, 26% some college, 46% college, 13% graduate degree  Control: 9% high school or less, 30% some college, 47% college, 14% graduate degree  Age  Intervention: 53% 18-24 years, 48% 25-29 years  Control: 54% 18-24 years, 47% 24-29 years  Sample size (overall response rate), follow-up: N=762 contributed data at 12 months (85% of those randomized)  N=741 contributed STI data at 12 months (82% of those randomized)  Intervention  N=370 contributed data at 12 months (83%)  N=363 contributed STI data as 12 months (82%)  Control  N=392 contributed data at 12 months (86%)  N=378 contributed STI data at 12 months (83%) | Description: see Mustanski et al (2013)  Technology: see Mustanski et al (2013)  Timing and duration: 7 modules had to be done at least 24 hours apart and took 2 hours to complete. These were followed by booster sessions at 3 and 6 months.  Target population: see Mustanski et al (2013)  Theoretical framework: see Mustanski et al (2013)  Development: see Mustanski et al (2013)  Provider organization: see Mustanski et al (2013)  Content: Online modules were based on situations and settings relevant to young MSM and used a variety of media and methods such as video, animation and games. Modules addressed, among other topics, condom use; triggers for unprotected sex; obtaining support; communication; the effects of mood, drug and alcohol abuse and sexual arousal; power dynamics in relationships; and the limits of serosorting. Users developed an HIV/STI prevention plan, and goals were suggested tailored to users’ baseline risks. 2 booster sessions reinforced learning, introduced new skills and provided an opportunity to review earlier goals.  Control: Online content similar to available didactic HIV prevention materials. Control was matched to the intervention in the number of modules and the requirement to participate in them over three sessions. At 3- and 6-month follow-up sessions (i.e., the same timing as intervention booster sessions) materials were reviewed again and information was provided on biomedical strategies. | Incident STI (N=733, 81% of those randomized):  Risk ratio (95% CI), intervention vs. control  Urethral chlamydia: 0.60 (0.12, 2.34)  Urethral gonorrhea: 0.35 (0.01, 4.33)  Rectal chlamydia: 0.61 (0.34, 1.06)  Rectal gonorrhea: 0.91 (0.40, 2.05)  Any STI: 0.60 (0.38, 0.95)  Any STI at month 12: 40% (95%CI=5%, 30%) lower in intervention arm, *p*=0.01  Paired analysis considered within-person changes in STIs while adjusting for between-arm differences in infection at baseline (N=729, 81% of those randomized)  Risk ratio=0.32 (95% CI=0.17, 0.60), *p*=0.0004  Condomless anal intercourse with casual partners in prior 3 months (N=757, 84% of those randomized)  Prevalence ratio at month 12: 0.83 (95% CI=0.70, 0.99), *p*=0.04  Estimated average effect over follow-up: Prevalence ratio 0.89, *p*=0.07  Percentage at month 12: Reported by 44% of control, 37% of intervention  Estimated average effect over follow-up: 11% (prevalence ratio=0.89, *p*=0.07)  Rates of self-reported incident HIV diagnoses  2%, with no differences between control (diagnosis rate=2.0%, 95% CI=0.84, 3.85) and intervention (2.3%, 95% CI=1.07, 4.45) |
| Reback et al (2019) [36] | Location – country (region): United States (Hollywood area of Los Angeles, California)  Target population: Non-treatment seeking methamphetamine-using MSM  Sampling: Street- and venue-based outreach, social media and dating app advertising, flyers, posters and participant referral. Eligible participants were non-treatment seeking methamphetamine-using MSM aged 18-65 years reporting condomless anal intercourse with non-primary male partner.  Sample size (overall response rate), baseline:  Overall: N=286 were randomized  Intervention: N=99  Control: N=94 randomized to comparison intervention (ineligible for this review) and N=93 randomized to control  Sexuality:  Overall: 67.1% gay identified, 32.9% non-gay identified  Intervention: 68.7% gay identified, 31.3% non-gay identified  Control: 62.4% gay identified, 37.6% non-gay identified  Gender identity: Not stated; eligible participants were MSM  Ethnicity:  Overall: 19.6% Caucasian/White, 43.7% African American/Black, 25.2% Hispanic/Latino, 11.5% Multiracial/other  Intervention: 16.2% Caucasian/White, 45.5% African American/Black, 29.3% Hispanic/Latino, 9.1% Multiracial/other  Control: 21.5% Caucasian/White, 43.0% African American/Black, 24.7% Hispanic/Latino, 10.8% Multiracial/Other  Socio-economic status:  Overall: 17.7% less than high school graduate/GED, 30.5% high school graduate/GED, 33.0% some college, 18.8%  Intervention: 17.5% less than high school graduate/GED, 33.0% high school graduate/GED, 27.8% some college, 21.7% college graduate  Control: 18.5% less than high school graduate/GED, 30.4% high school graduate/GED, 37.0% some college, 14.1% college graduate  Age:  Overall: mean 41.5 years (SD=10.9)  Intervention: mean 41 years (SD=11.1)  Control: mean 41.4 years (SD=10.2)  Sample size (overall response rate), follow-up:  Overall  8-week follow-up: N=237 (83%)  3-month follow-up: N=251 (88%)  6-month follow-up: N=240 (84%)  9-month follow-up: N=255 (89%)  Intervention  8-week follow-up: N=82 (83%)  3-month follow-up: N=82 (83%)  6-month follow-up: N=83 (84%)  9-month follow-up: N=85 (86%)  Control  8-week follow-up: N=79 (85%)  3-month follow-up: N=83 (89%)  6-month follow-up: N=78 (84%)  9-month follow-up: N=84 (90%) | Description: Text message-based intervention to reduce substance use and HIV risk  Technology: Text messaging  Timing and duration: 5 messages per day for 8-weeks, delivered at peak hours of high-risk activities (Mon and Tues 12-8pm, Wed and Thurs 12pm-1am, Fri 12pm-2am, Sat 3:30pm-2am, and Sun 3:30pm-12am). Weekly self-monitoring assessments.  Target population: Out-of-treatment methamphetamine-using MSM  Theoretical framework: Text message content was based on social support theory, social cognitive theory and the health belief model, which the authors described as complementary theories. Messages aimed to increase knowledge, and the authors’ description suggested they might also aim to increase self-efficacy. A brief weekly text-based assessment asking about methamphetamine use and HIV sexual behaviors in the past 7 days aimed to increase self-monitoring.  Content: TXT-Auto aimed to reduce substance use and HIV risk by decreasing methamphetamine use, sex during methamphetamine use and condomless anal intercourse. A baseline survey assessed the user’s risk profile in relation to HIV status, ART adherence, drug use and sexual behaviors. Users then received 5 automated scripted text messages per day, which included both general messages and messages tailored to their risk profile. A brief weekly text-based assessment asking about methamphetamine use and HIV sexual behaviors in the past 7 days aimed to increase self-monitoring.  Control: Participants in the assessment-only condition received the same welcome message and brief weekly text-based assessments on their methamphetamine use and HIV sexual behaviors in the past 7 days, and follow-up appointment reminders. This provided an ‘attentional control.’ | Days of methamphetamine use  Interaction effects-condition X time point: Coef. (95% CI)  TXT-Auto*Time: 0.01 (-0.04, 0.06)  Constant: 0.08 (-0.22, 0.38)  Episodes of sex while on methamphetamine  Interaction effects-condition X time point: Coef. (95% CI)  TXT-Auto*Time: -0.05 (-0.11, 0.009), significant at *p*≤0.10  Constant: -0.10 (-0.39, 0.19)  Episodes of CAI with male partner (insertive or receptive)  Interaction effects-condition X time point: Coef. (95% CI)  main partners: TXT-Auto*Time: -0.16 (-0.31, 0.003), *p*≤0.10  casual partners: TXT-Auto*Time: -0.01 (-0.06, 0.03)  anonymous partners: TXT-Auto*Time: -0.05 (-0.10, 0.003), p*≤*0.10  Partners for transactional sex: TXT-Auto*Time: -0.03 (-0.18, 0.11) |
| Rosser et al (2010) [37] | Location – country (region): United States  Target population: MSM  Sampling: Recruited via banner ads on two of the largest gay website in the US, and via emails to past research participants. Eligible participants were male US residents aged 18 years or older, with recent history of unprotected anal intercourse with at least one other man.  Sample size (overall response rate), baseline: Overall: N=650 completed baseline survey and were randomized (63% of those eligible)  Intervention: N=337 began intervention  Control: N=313 began control survey  Sexuality:  Overall: 91.4% homosexual/gay/same gender loving, 8.6% bisexual/straight/other  Intervention: 90.8% homosexual/gay/same gender loving; 9.2% bisexual/straight/other  Control: 92.0% homosexual/gay/same gender loving, 8.0% bisexual/straight/other  Gender identity: Not stated; eligible participants were MSM  Ethnicity:  Overall: 68.2% White, 6.3% Black or African American, 15.1% Latino/Spanish/other, 3.5% Asian, 6.9% Other  Intervention: 71.2% White, 5.3% Black or African American, 13.1% Latino/Spanish/other, 2.7% Asian, 7.7% Other  Control: 64.9% White, 7.4% Black or African American, 17.3% Latino/Spanish/other, 4.5% Asian, 6.1% Other  Socio-economic status:  Education  Overall: 7.9% less than high school or high school graduate, 34.3% some college education, 22.6% college degree, 35.2% graduate/professional school  Intervention: 8.6% less than high school or high school graduate, 34.7% some college education, 22.0% college degree, 34.7% graduate/professional school  Control: 7.0% less than high school or high school graduate, 33.9% some college education, 23.3% college degree, 35.8% graduate/professional school  Annual income  Overall: 18.6% less than $20,000; 19.5% $20,000-$31,999; 18.0% $32,000-$44,999; 19.1% $45,000-$64,999; 20.5% greater than $65,000; 4.3% refuse to answer  Intervention: Annual income: 19.3% less than $20,000; 18.1% $20,000-$31,999; 17.8% $32,000-$44,999; 20.2% $45,000-$64,999; 20.5% greater than $65,000; 4.2% refuse to answer  Control: Annual income: 17.9% less than $20,000; 21.1% $20,000-$31,999; 18.2% $32,000-$44,999; 17.9% $45,000-$64,999; 20.5% greater than $65,000; 4.5% refuse to answer  Residence  Overall: Residence: 16.1% rural or small town, 16.4% medium-sized city, 24.0% suburb of a large-sized city, 43.5% downtown or central district of a large-sized city  Intervention: Residence: 17.1% rural or small town, 18.0% medium-sized city, 24.0% suburb of a large-sized city, 41.0% downtown or central district of a large-sized city  Control: Residence: 15.1% rural or small town, 14.7% medium-sized city, 24.0% suburb of a large-sized city, 46.2% downtown or central district of a large-sized city  Age:  Overall: 23.5% ages 18-25; 34.5% ages 26-35; 27.7% ages 36-45; 14.3% older than age 45  Intervention: 24.0% ages 18-25, 33.5% ages 26-35; 27.3% ages 36-45, 15.1% older than 45  Control: 23.0% ages 18-25, 35.5% ages 26-35, 28.1% ages 36-45, 15.1% older than 45  Sample size (overall response rate), follow-up:  Overall  Completed post-treatment/post-control survey: N=587 (90%)  Completed 3-month survey: N=560 (86%)  Completed 12-month survey: N=554 (85%)  Intervention  Completed post-treatment survey: N=276 (82%)  Completed 3-month survey: N=267 (79%)  Completed 12-month survey: N=276 (82%)  Control  Completed post-control survey: N=311 (99%)  Completed 3-month survey: N=293 (94%)  Completed 12-month survey: N=278 (89%) | Description: Modular HIV prevention intervention to reduce unprotected anal intercourse  Technology: Internet  Timing and duration: Multi-module intervention was to be completed over a 7-day period  Target population: MSM  Theoretical framework: Guided by the sexual health model, which posits that people are more likely to make sexually healthy decisions when they themselves are sexually healthy. Intervention addressed the following aspects of the model: (1) mental and emotional health, (2) physical health, (3) intimacy, (4) relationships, (5) sexuality, and (6) spirituality. Content covered other specified topics but their relationship to the sexual health model and to the intervention was not clear. Based on the authors’ description, the theory underpinning the intervention seemed to be that addressing aspects of broader sexual health would support safer sexual health decision-making.  Content: Sexpulse was a flexible intervention and incorporated video segments, interactive text and animations. Examples of modules included a ‘hot sex calculator’ demonstrating decision-making, a virtual gym where users could explore body image concerns, an online chat simulation to explore evasive and ambiguous communication and a ‘reflective journey’ exploring past experiences, long-term goals and spirituality. The intervention addressed a range of topics including mental, emotional and physical health; intimacy; relationships; sexuality; and spirituality. Modules were supplemented with FAQs, virtual peers sharing their experiences, cartoons and interactive polls.  Control: Waitlist null control; participants randomized to the control arm completed an additional sexual health survey between baseline and post-intervention assessments. | Unprotected anal intercourse with male partner  At 3-month follow-up  Unadjusted: Incident rate ratio=0.832 (95% CI=0.691, 1.000), *p*=0.050  Adjusted: Incident rate ratio=0.8444 (95% CI=0.704, 1.1013), *p*=0.068  At 12-month follow-up  Unadjusted: Incident rate ratio 0.998 (95% CI=0.952, 1.046), *p*=0.921  Adjusted: Incident rate ratio 0.998 (95% CI=0.952, 1.046), *p*=0.937 |
| Schonnesson et al (2016) [38] | Location – country (region): Sweden  Target population: Swedish MSM  Sampling: Recruited via banners on Swedish website popular among LGBTQ people. Eligible participants were males 15 years or older who were fluent in Swedish and reported sex with a man in the prior 12 months  Sample size (overall response rate), baseline:  N=112 completed pre-test questionnaire and were randomized (83% of those eligible)  Intervention: N=58  Control: N=54  Sexuality: 93% gay  Gender identity: Not stated; eligible participants were MSM  Ethnicity: Not stated  Socio-economic status:  Education: 88% completed more than high school  Employment: 79% employed; remainder unemployed or on sick leave  Residence: 55% lived in a city; others lived in a town or in the countryside  Age: Mean 32 years (SD=12.09)  Sample size (overall response rate), follow-up:  30-day questionnaire:  Overall: N=55 (49% of those randomized)  Intervention: N=25 (43% of those randomized)  Control: N=33 (61% of those randomized) | Description: Online modular HIV risk reduction intervention for MSM  Technology: See Bowen (2008)  Timing and duration: Three modules each contained two 20-minute sessions. Sessions had to be completed 24-48 hours apart.    Target population: Sexually active, internet-using MSM  Theoretical framework: See Bowen (2008)  Content: Module content included information tailored for rural MSM and was presented as conversations between gay men. It used the Swedish language, including reflecting language expressions, and content was consistent with Swedish health care and HIV programs. Dialogue was interspersed with interactive activities and graphics. The first module featured a conversation between an HIV-positive gay man who represented an ‘expert’ and an ‘inexperienced’ HIV-negative gay man who had recently had a high-risk sexual encounter, primarily addressing HIV prevention during sex and living with HIV. It and also included information about STIs and about the Swedish Communicable Disease Act. This module featured links to websites with further information.  The second module featured a conversation between 5 gay male friends with one representing the user and aimed to increase motivation, and a third module targeting behavioral skills in a similar format was introduced. Both allowed users to print a summary of their responses to interactive components. The ‘motivation’ module helped users identify reasons for not using condoms and ways to address these to support the user’s pursuit of their life goals. The ‘behavior’ module addressed approaches for reducing sexual risk with partners met online or in a bar.  Control: Waitlist control. Those randomized to the control group waited 30 days, then completed the posttest questionnaire, then could access the intervention. | Outcome data for sexual risk behaviors not analyzed due to low sample numbers |
