# Supplementary material for: eHealth Interventions to Address HIV and Other Sexually Transmitted Infections, Sexual Risk Behavior, Substance Use, and Mental Ill-health in Men Who Have Sex With Men: Systematic Review and Meta-analysis
Source: JMIR Public Health Surveill. 2022 Apr 6;8(4):e27061. doi: 10.2196/27061 (PMC9021948; doi:10.2196/27061)
Supplement: Multimedia Appendix 3 [file publichealth_v8i4e27061_app3.docx]

**Multimedia Appendix 3.** Study-level risk of bias judgments

Items used to appraise studies were as follows:

- Sequence generation: was the allocation sequence adequately generated?
- Allocation concealment: was the allocation adequately concealed?
- Blinding of participants/personnel: was knowledge of intervention allocation adequately prevented during the study regarding participants and study personnel?
- Blinding of outcome assessors: was knowledge of intervention allocation adequately prevented during the study regarding outcome assessors?
- Complete outcome data: were complete data for each outcome reported, and, if not were adequate reasons for incomplete outcome data provided?
- No selective outcome reporting: were the findings of the study free of selective outcome reporting?
- Reduced other sources of bias: did authors aim to reduce other forms of bias that might have entered the study?

Bauermeister 2019 [26]

| *Item* | *Reviewer judgement* | *Description* |
| --- | --- | --- |
| **Sequence generation** | Yes | Used pseudo-random number generator with permutated blocks |
| **Allocation concealment** | Yes | Allocation conducted automatically online |
| **Blinding of participants/personnel** | No | Study participants were blinded but analysts were not |
| **Blinding of outcome assessors** | Yes | Outcomes were self-report and participants were blinded |
| **Complete outcome data** | Yes |  |
| **No selective outcome reporting** | No | Complete for primary outcomes but not for relevant secondary outcomes specified in protocol (psychological well-being, substance use) |
| **Reduced other sources of bias** | Yes | Recruited nationally |

Bowen 2008 [39]

| *Item* | *Reviewer judgement* | *Description* |
| --- | --- | --- |
| **Sequence generation** | Not stated |  |
| **Allocation concealment** | Yes | Randomization appears to have taken place automatically online |
| **Blinding of participants/personnel** | Not stated | Does not state whether participants or personnel were blinded to intervention allocation |
| **Blinding of outcome assessors** | Not stated | All outcomes were self-reported; information on participant blinding was not stated |
| **Complete outcome data** | No | Attrition varied by arm and was <70% overall |
| **No selective outcome reporting** | No | No concrete estimates for between-group differences presented |
| **Reduced other sources of bias** | No | No clear evidence of efficacy presented; no control group; no accounting for high rates of attrition |

Carpenter 2010 [27]

| *Item* | *Reviewer judgement* | *Description* |
| --- | --- | --- |
| **Sequence generation** | Yes | Computerized algorithm used random number tables to balance groups by race and ethnicity |
| **Allocation concealment** | Yes | All study procedures took place online |
| **Blinding of participants/personnel** | No | Participants were blinded but study personnel were not |
| **Blinding of outcome assessors** | Yes | All outcomes were self-reported and participants were blinded |
| **Complete outcome data** | No | High attrition across all arms, >40% |
| **No selective outcome reporting** | No | Use of MANOVA precludes presentation of all relevant group by time effects for individual outcomes; use of log transformation and MANOVA precludes complete presentation of results |
| **Reduced other sources of bias** | Yes | Sample recruited from a variety of US cities |

Cheng 2019 [28]

| *Item* | *Reviewer judgement* | *Description* |
| --- | --- | --- |
| **Sequence generation** | Yes | Computer algorithm used to randomize participants |
| **Allocation concealment** | Yes | Allocation appears to have been conducted automatically online |
| **Blinding of participants/personnel** | No | Participants were not blinded |
| **Blinding of outcome assessors** | No | Outcomes were self-reported and participants were not blinded |
| **Complete outcome data** | Yes |  |
| **No selective outcome reporting** | Yes |  |
| **Reduced other sources of bias** | Yes | Appears to have recruited participants from throughout China |

Chiou 2020 [29]

| *Item* | *Reviewer judgement* | *Description* |
| --- | --- | --- |
| **Sequence generation** | Yes | Rolled dice and corresponded to random number table |
| **Allocation concealment** | Yes |  |
| **Blinding of participants/personnel** | Yes |  |
| **Blinding of outcome assessors** | Yes |  |
| **Complete outcome data** | Yes |  |
| **No selective outcome reporting** | Yes |  |
| **Reduced other sources of bias** | Yes | Recruited participants from throughout the country |

Christensen 2013 [30]

| *Item* | *Reviewer judgement* | *Description* |
| --- | --- | --- |
| **Sequence generation** | Yes | Online data collection software automatically generated random allocation sequence and assigned participants to study condition |
| **Allocation concealment** | Yes | Allocation conducted by online data collection software |
| **Blinding of participants/personnel** | No | Researchers were blind to allocation at enrolment but some were subsequently unblinded to avoid participant re-enrolment. Does not state whether participants were blinded. |
| **Blinding of outcome assessors** | Not stated | All outcomes were self-reported; does not state whether participants were blinded |
| **Complete outcome data** | No | Retention was <70% |
| **No selective outcome reporting** | No | No direct estimate on unprotected anal intercourse provided, nor was one calculable |
| **Reduced other sources of bias** | No | Analytic methods used to estimate mediational pathways are biased, including use of residualized change scores |

Davidovich 2006 [31]

| *Item* | *Reviewer judgement* | *Description* |
| --- | --- | --- |
| **Sequence generation** | Not stated | Only states that participants were randomly assigned to one of three conditions |
| **Allocation concealment** | Yes | Randomization appears to have taken place automatically online |
| **Blinding of participants/personnel** | No | Participants were blinded but study personnel were not |
| **Blinding of outcome assessors** | Yes | All outcomes are self-reported and participants were blinded |
| **Complete outcome data** | No | High levels of attrition across all conditions; does not appear balanced by arm |
| **No selective outcome reporting** | Yes | All stated outcomes were reported |
| **Reduced other sources of bias** | Yes | Used multiple methods of recruitment to obtain a diverse sample |

Hirshfield 2019 [32]

| *Item* | *Reviewer judgement* | *Description* |
| --- | --- | --- |
| **Sequence generation** | Not stated |  |
| **Allocation concealment** | Yes | Randomization took place automatically online |
| **Blinding of participants/personnel** | Not stated |  |
| **Blinding of outcome assessors** | Not stated | Outcomes were self-report; doesn’t state whether participants were blinded |
| **Complete outcome data** | Yes |  |
| **No selective outcome reporting** | No | Protocol (Hirshfield 2016) specifies outcomes which are not reported in this paper |
| **Reduced other sources of bias** | Yes | Recruited nationally and from multiple sources |

Milam 2016 [33]

| *Item* | *Reviewer judgement* | *Description* |
| --- | --- | --- |
| **Sequence generation** | Not stated |  |
| **Allocation concealment** | Yes |  |
| **Blinding of participants/personnel** | No | Participants and study coordinator were not blinded. |
| **Blinding of outcome assessors** | Yes | Clinicians and the adjudication committee verifying newly diagnosed STIs were both blinded |
| **Complete outcome data** | Yes | >70% retention in both arms, proportions roughly balanced |
| **No selective outcome reporting** | No | Estimates for secondary endpoints not presented in sufficient detail to evaluate magnitude of change |
| **Reduced other sources of bias** | No | Presentation of as-treated and completer analyses do not yield unbiased estimates of treatment effect |

Mustanski 2013 [34]

| *Item* | *Reviewer judgement* | *Description* |
| --- | --- | --- |
| **Sequence generation** | Yes | Participants randomized by computerized algorithm; groups stratified by race |
| **Allocation concealment** | Yes | Randomization was done using a computer algorithm and eligibility was assessed online |
| **Blinding of participants/personnel** | Yes | Both participants and staff with direct participant contact were blinded to patient allocation |
| **Blinding of outcome assessors** | Yes | Outcomes were self-reported by participants, who were blinded |
| **Complete outcome data** | Yes | Attrition was around 70% for both arms |
| **No selective outcome reporting** | Yes | All estimates presented |
| **Reduced other sources of bias** | Yes |  |

Mustanski 2018 [35]

| *Item* | *Reviewer judgement* | *Description* |
| --- | --- | --- |
| **Sequence generation** | Yes | Randomized used six permuted blocks of size four; groups stratified by race and by HIV testing site |
| **Allocation concealment** | Yes | Randomization conducted via an eHealth platform and appears to have taken place online |
| **Blinding of participants/personnel** | Yes | Participants and the study staff who had contact with participants were both blinded to allocation |
| **Blinding of outcome assessors** | Yes | Primary outcome is laboratory-based |
| **Complete outcome data** | Yes | Low and balanced attrition between arms |
| **No selective outcome reporting** | No | The protocol listed six secondary outcomes (HIV knowledge, HIV motivation and behavioral skills, condom errors, health protective communication, PrEP intentions and use, and intervention acceptability) that are not reported herein |
| **Reduced other sources of bias** | Yes | Recruitment used multiple methods and targeted different US regions, in addition to local and national advertising. Participants who withdrew or moved out of the country were not excluded from analysis. |

Reback 2019 [36]

| *Item* | *Reviewer judgement* | *Description* |
| --- | --- | --- |
| **Sequence generation** | Not stated |  |
| **Allocation concealment** | Not stated |  |
| **Blinding of participants/personnel** | No | Neither participants nor study personnel were blinded |
| **Blinding of outcome assessors** | No | Outcomes are self-reported and participants were not blinded |
| **Complete outcome data** | Yes | Attrition low and consistent across arms |
| **No selective outcome reporting** | Yes | All outcomes reported |
| **Reduced other sources of bias** | Yes | Used multiple methods of recruitment with the aim of recruiting a diverse sample |

Rosser 2010 [37]

| *Item* | *Reviewer judgement* | *Description* |
| --- | --- | --- |
| **Sequence generation** | Yes | Computer algorithm used to randomly assign participants to a study arm |
| **Allocation concealment** | Yes |  |
| **Blinding of participants/personnel** | No | Participants were not blinded; does not state whether personnel were blinded |
| **Blinding of outcome assessors** | No | Outcomes were self-reported and participants were not blinded |
| **Complete outcome data** | Yes | Attrition roughly balanced between arms, >70% in each arm |
| **No selective outcome reporting** | Yes | All outcomes reported as described |
| **Reduced other sources of bias** | Yes | Used multiple recruitment methods and did not target one single geographic region |

Schonnesson 2016 [38]

| *Item* | *Reviewer judgement* | *Description* |
| --- | --- | --- |
| **Sequence generation** | Not stated |  |
| **Allocation concealment** | Yes | Randomization appears to have taken place automatically online |
| **Blinding of participants/personnel** | No | Neither participants nor study personnel were blinded |
| **Blinding of outcome assessors** | No | All outcomes were self-reported and participants were not blinded |
| **Complete outcome data** | No | Attrition uneven, and high across both arms |
| **No selective outcome reporting** | No | Only the outcome of anal sex index (casual partner) was examined because the sample sizes for the other three sex risk variables were too small |
| **Reduced other sources of bias** | No | Change scores were computed for primarily outcome variables and intermediate cognitive outcome variables by subtracting pretest from posttest scores. |

GRADE assessments

| **Certainty assessment** | | | | | | | **№ of patients** | | **Effect** | | **Certainty** | **Importance** |
| --- | --- | --- | --- | --- | --- | --- | --- | --- | --- | --- | --- | --- |
| **№ of studies** | **Study design** | **Risk of bias** | **Inconsistency** | **Indirectness** | **Imprecision** | **Other considerations** | **eHealth** | **control** | **Relative (95% CI)** | **Absolute (95% CI)** |  |  |
| **Drug use (short-term)** | | | | | | | | | | | | |
| 2 | randomized trials | serious ^a^ | very serious ^b^ | not serious | very serious ^c^ | none | Estimates could not be pooled due to high heterogeneity and few effect sizes. | | | | ⨁◯◯◯ VERY LOW | CRITICAL |
| **Drug use (mid-term)** | | | | | | | | | | | | |
| 1 | randomized trials | serious ^a^ | not serious | not serious | very serious ^c^ | none | Estimates drew from one study, Reback 2018. This study did not provide evidence of effectiveness. | | | | ⨁◯◯◯ VERY LOW | CRITICAL |
| **HIV infections (short-term)** | | | | | | | | | | | | |
| 1 | randomized trials | not serious | not serious | not serious | very serious ^c^ | none | Estimates drew from one study, Chiou 2020. This study did not provide evidence of effectiveness. | | | | ⨁⨁◯◯ LOW | CRITICAL |
| **HIV infections (mid-term)** | | | | | | | | | | | | |
| 1 | randomized trials | serious ^d^ | not serious | not serious | very serious ^c^ | none | Estimate drew from one study, Mustanski 2018. This study did not provide evidence of effectiveness. | | | | ⨁◯◯◯ VERY LOW | CRITICAL |
| **Sexually transmitted infections (short-term)** | | | | | | | | | | | | |
| 2 | randomized trials | very serious ^a,d^ | not serious | not serious | very serious ^c^ | none | A pooled estimate of both studies suggested a non-significant increase in STIs as a result of interventions (*d*=0.17, 95% CI [-0.18, 0.52]). | | | | ⨁◯◯◯ VERY LOW | CRITICAL |
| **Sexually transmitted infections (mid-term)** | | | | | | | | | | | | |
| 1 | randomized trials | serious ^d^ | not serious | not serious | not serious | none | Estimates drew from one study, Mustanski 2018. The pooled outcome of 'any STI' suggested a significant reduction in risk of STIs (RR=0.32, 95% CI [0.40, 0.83]). | | | | ⨁⨁⨁◯ MODERATE | CRITICAL |
| **Sexual risk behavior (short-term)** | | | | | | | | | | | | |
| 8 | randomized trials | very serious ^a,d^ | serious ^e^ | not serious | not serious | publication bias strongly suspected ^f^ | Pooled estimates suggested a non-significant decrease in sexual risk behavior (*d*=-0.14, 95% CI [-0.30, 0.03] ). | | | | ⨁◯◯◯ VERY LOW | CRITICAL |
| **Sexual risk behavior (mid-term)** | | | | | | | | | | | | |
| 6 | randomized trials | very serious ^a,d^ | not serious | not serious | not serious | none | Pooled estimates suggested a significant reduction in sexual risk behavior (*d*=-0.12, 95% CI [-0.19, -0.05] ). | | | | ⨁⨁◯◯ LOW | CRITICAL |
