# Supplementary material for: eHealth Interventions to Address HIV and Other Sexually Transmitted Infections, Sexual Risk Behavior, Substance Use, and Mental Ill-health in Men Who Have Sex With Men: Systematic Review and Meta-analysis
Source: JMIR Public Health Surveill. 2022 Apr 6;8(4):e27061. doi: 10.2196/27061 (PMC9021948; doi:10.2196/27061)

**Multimedia Appendix 4.** Forest plots for sexually transmitted infection outcomes

Short-term outcomes: incident sexually transmitted infections


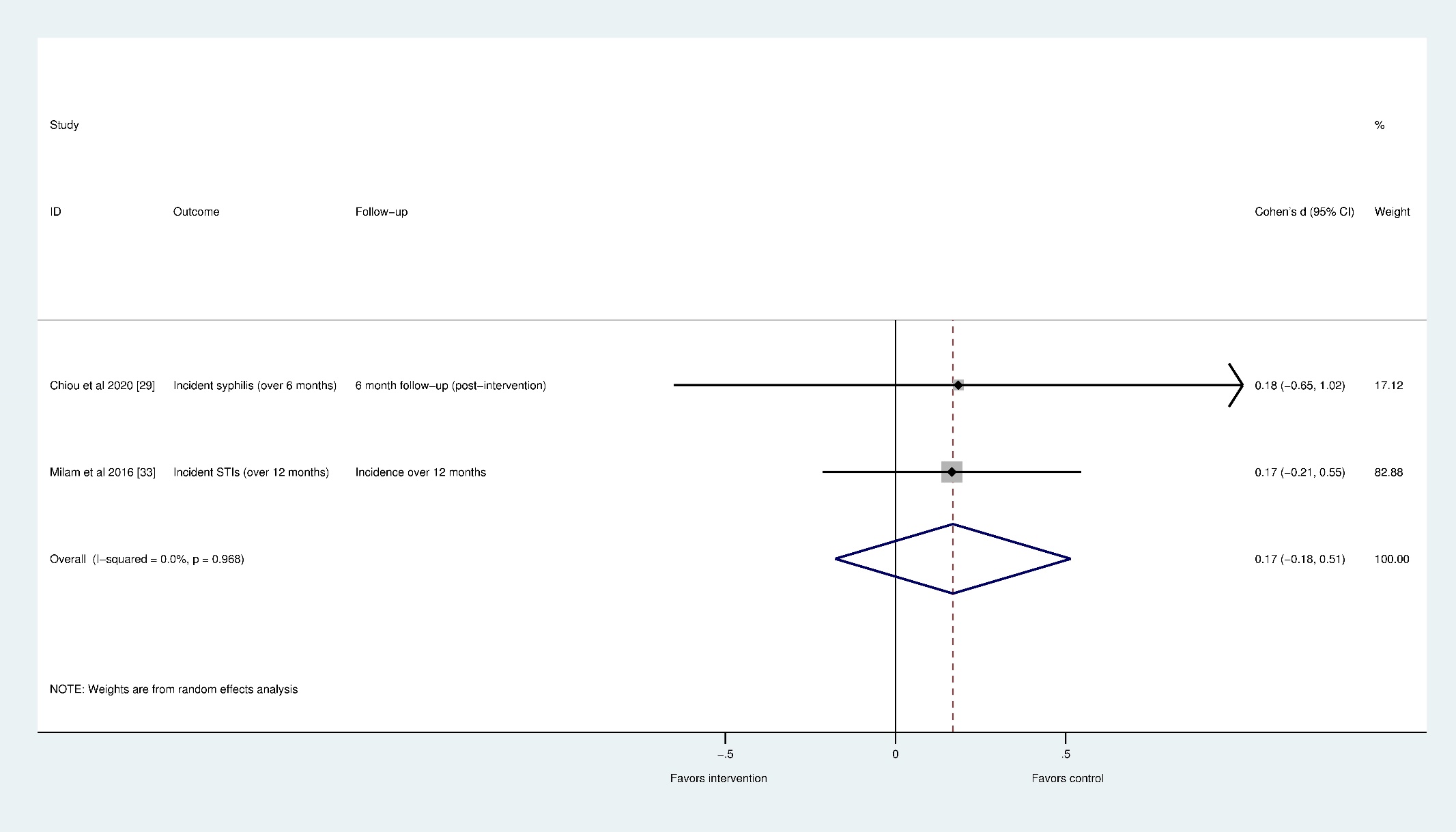


Mid-term outcomes: sexually transmitted infections


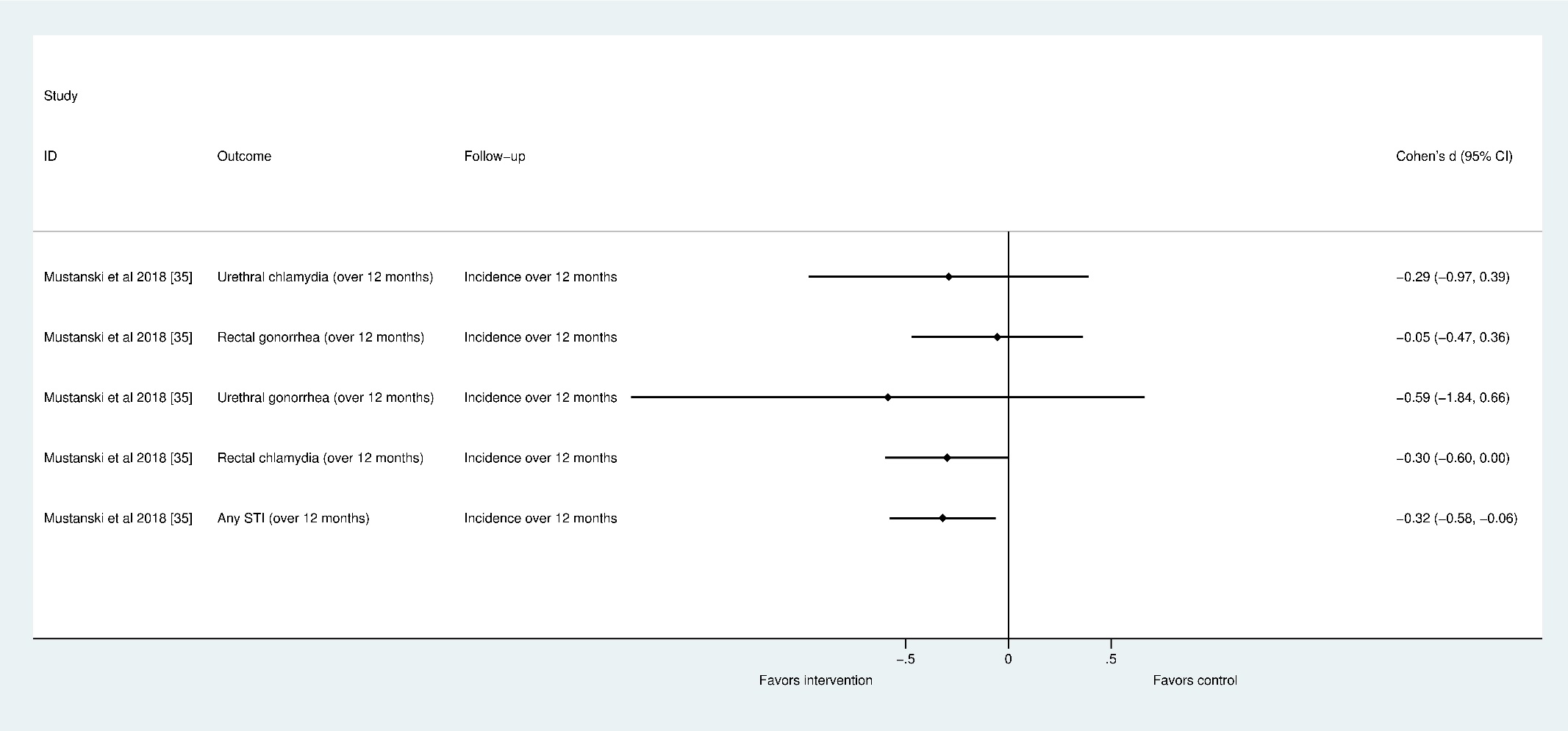

Supplement: Multimedia Appendix 4 [file publichealth_v8i4e27061_app4.docx]
